# Long-term associations between amyloid positron emission tomography, sex, apolipoprotein E and incident dementia and mortality among individuals without dementia: hazard ratios and absolute risk

**DOI:** 10.1093/braincomms/fcac017

**Published:** 2022-02-02

**Authors:** Clifford R. Jack, Terry M. Therneau, Emily S. Lundt, Heather J. Wiste, Michelle M. Mielke, David S. Knopman, Jonathan Graff-Radford, Val J. Lowe, Prashanthi Vemuri, Christopher G. Schwarz, Matthew L. Senjem, Jeffrey L. Gunter, Ronald C. Petersen

**Affiliations:** 1 Department of Radiology, Mayo Clinic, Rochester, MN, USA; 2 Department of Quantitative Health Sciences, Mayo Clinic, Rochester, MN, USA; 3 Department of Neurology, Mayo Clinic, Rochester, MN, USA; 4 Department of Nuclear Medicine, Mayo Clinic, Rochester, MN, USA

**Keywords:** amyloid PET, dementia, mortality, APOE, sex

## Abstract

Dementia and mortality rates rise inexorably with age and consequently interact. However, because of the major logistical difficulties in accounting for both outcomes in a defined population, very little work has examined how risk factors and biomarkers for incident dementia are influenced by competing mortality. The objective of this study was to examine long-term associations between amyloid PET, APOE ɛ4, sex, education and cardiovascular/metabolic conditions, and hazard and absolute risk of dementia and mortality in individuals without dementia at enrolment. Participants were enrolled in the Mayo Clinic Study of Aging, a population-based study of cognitive ageing in Olmsted County, MN, USA. All were without dementia and were age 55–92 years at enrolment and were followed longitudinally. Predictor variables were amyloid PET, APOE ɛ4 status, sex, education, cardiovascular/metabolic conditions and age. The main outcomes were incident dementia and mortality. Multivariable, multi-state models were used to estimate mortality and incident dementia rates and absolute risk of dementia and mortality by predictor variable group. Of the 4984 participants in the study, 4336 (87%) were cognitively unimpaired and 648 (13%) had mild cognitive impairment at enrolment. The median age at enrolment was 75 years; 2463 (49%) were women. The median follow-up time was 9.4 years (7.5 years after PET). High versus normal amyloid (hazard ratio 2.11, 95% confidence interval 1.43–2.79), APOE ɛ4 (women: hazard ratio 2.24, 95% confidence interval 1.80–2.77; men: hazard ratio 1.37, 95% confidence interval 1.09–1.71), older age and two additional cardiovascular/metabolic conditions (hazard ratio 1.37, 95% confidence interval 1.22–1.53) were associated with the increased hazard of dementia (all *P* < 0.001). Among APOE ɛ4 carriers with elevated amyloid, remaining lifetime risk of dementia at age 65 years was greater in women [74% (95% confidence interval 65–84%) high and 58% (95% confidence interval 52–65%) moderate amyloid], than men [62% (95% confidence interval 52–73%) high and 44% (95% confidence interval 35–53%) moderate amyloid]. Overall, the hazard and absolute risk of dementia varied considerably by predictor group. The absolute risk of dementia associated with predictors characteristic of Alzheimer’s disease was greater in women than men while at the same time the combination of APOE ɛ4 non-carrier with normal amyloid was more protective in women than men. This set of findings may be attributed in part to different biological effects and in part to lower mortality rates in women.

## Teaser

The objective of this study was to examine long-term associations between amyloid PET, APOE ɛ4, sex, education and cardiovascular/metabolic conditions, and hazard and absolute risk of dementia and mortality in individuals without dementia at enrolment. Overall, the hazard and absolute risk of dementia varied considerably by predictor group.

## Graphical Abstract

**Figure:**
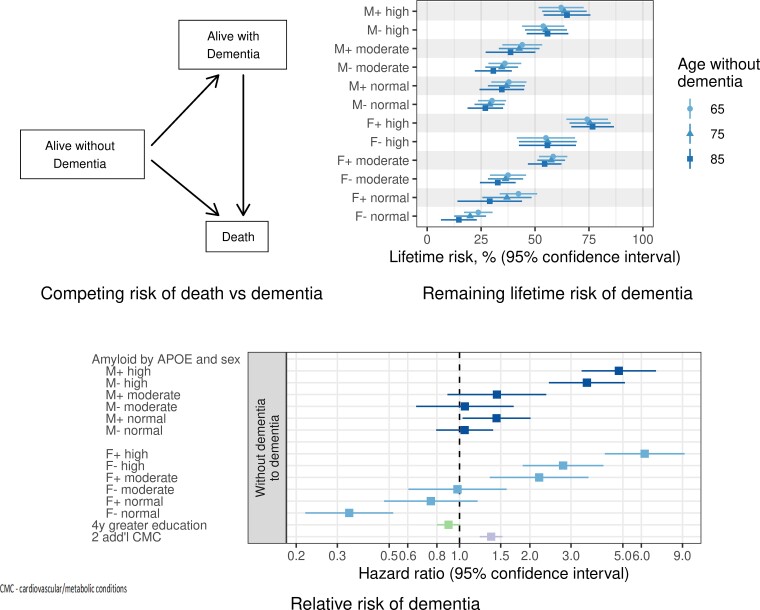

## Introduction

Dementia in elderly individuals is typically due to combinations of ageing-related brain pathologies which often, but not necessarily, include Alzheimer’s disease.^1–3^ Alzheimer’s disease is defined by the presence of both β-amyloid plaques and tau neurofibrillary tangles,^4^ which can be ascertained *in vivo* by PET imaging or biofluid biomarkers.^5^

The objective of this study was to examine the long-term relationships between amyloid PET, APOE, sex, education and cardiovascular/metabolic conditions (CMC), and two clinically meaningful outcomes—incident dementia and mortality. Prior work has demonstrated that the predictor variables we evaluated—amyloid PET, APOE, age, sex, education and CMC—are related to the risk of dementia.^6–15^ These predictors were accurately captured in this study cohort.

Because both incident dementia and mortality increase with advancing age, failure to account for the competing risk of death impacts interpretation of the effects of risk factors and biomarkers on dementia incidence. The Mayo Clinic Study of Aging (MCSA) is a longitudinal observational study that is uniquely positioned to address this.

First, we were able to capture the two primary outcomes in both active participants and those who discontinued in person follow-up visits. In the latter group, clinical status could be determined by review of their medical records, owing to the unique design of the Rochester Epidemiology Project. Longer observation periods lead to greater cumulative withdrawal which in turn introduces greater selection bias in the remaining cohort. Therefore, this unique feature mitigated selection bias.^16^

Second, because we ascertained both incident dementia and death in the same defined population, we were able to calculate absolute risks for dementia, which are more interpretable, and more relevant to patients, than relative rates [i.e. hazard ratios (HRs)], the norm in Alzheimer’s biomarkers studies. Further, we applied analytic methods—multivariable, multi-state models—that are unique in their ability to portray competing outcomes.

A third distinguishing feature was a long-term follow-up after enrolment (median 9.4 years overall, 7.5 years post amyloid PET, maximum 15.7 years). Much of the existing literature relating amyloid PET to clinical outcomes in individuals without dementia has focused on cognitive change over short-to-medium observation periods (∼1–5 years).^7,17–27^ However, neuropathological changes leading to dementia evolve slowly^8^ and long-term follow-up is needed to fully capture associations between outcomes and upstream predictor variables.

## Materials and methods

### Participants

This study was approved by the Mayo Clinic and the Olmsted Medical Center Institutional Review Boards. All participants provided written informed consent.

All study participants were enrolled in the MCSA, a population-based epidemiological cognitive ageing study among a stratified random sample of a geographically defined population, Olmsted County, MN, USA (see Supplementary material).^28^ A clinical diagnosis was determined for each participant at enrolment and for all subsequent visits using clinical criteria alone.^28^ All participants in the present study were 55 years of age or older and without dementia at enrolment, including both mild cognitive impairment (MCI)^29^ and cognitively unimpaired (defined as not MCI and without dementia)^30^ participants.

### Predictor variables

Predictor variables were age, sex, education, APOE genotype, the first available amyloid PET and the first available composite CMC score. CMC score is the sum of the presence or absence of seven vascular-health-related conditions. A higher score indicates worse cardiometabolic health (see Supplementary material).^31,32^ All participants had an initial study visit including at minimum a clinical evaluation and blood draw between November 2004 and September 2020. MCSA participants without a medical contraindication were invited to participate in imaging studies.

Amyloid PET imaging was performed with Pittsburgh Compound B^33^ using previously described methods (see Supplementary material).^34^ The continuous range of amyloid PET values was divided into normal, moderately elevated (referred to as *moderate*) and highly elevated (referred to as *high*) ranges on the Centiloid scale.^35,36^ The cut point separating normal and moderate amyloid was Centiloid 22 [standardized uptake value ratio (SUVR) 1.48] which is the value beyond which rates of amyloid PET reliably increase.^34^ The value separating moderate from high amyloid was Centiloid 68 (SUVR 2.0) which corresponds to the global maxima of the amyloid PET SUVR by delta amyloid curve.^37,38^

### Outcomes

The two main outcomes were incident dementia, which was based on DSM IV criteria,^30^ and mortality. Participants were followed from enrolment through all MCSA visits until an event or censoring occurred (both incident dementia and mortality occurred in some individuals). Outcomes in study participants who had previously discontinued in person study follow-up visits were ascertained through semiannual reviews of the electronic medical record^39^ using the Rochester Epidemiology Project medical records-linkage system (see Supplementary material).^40^

### Statistical analysis

#### Overall death and incident dementia rates by age and sex

This analysis was performed to facilitate comparisons with epidemiological literature and was based on a person-years analysis, using Poisson models to calculate confidence intervals (CIs) and *P*-values.^41^

#### Multi-state model

**Figure 1:**
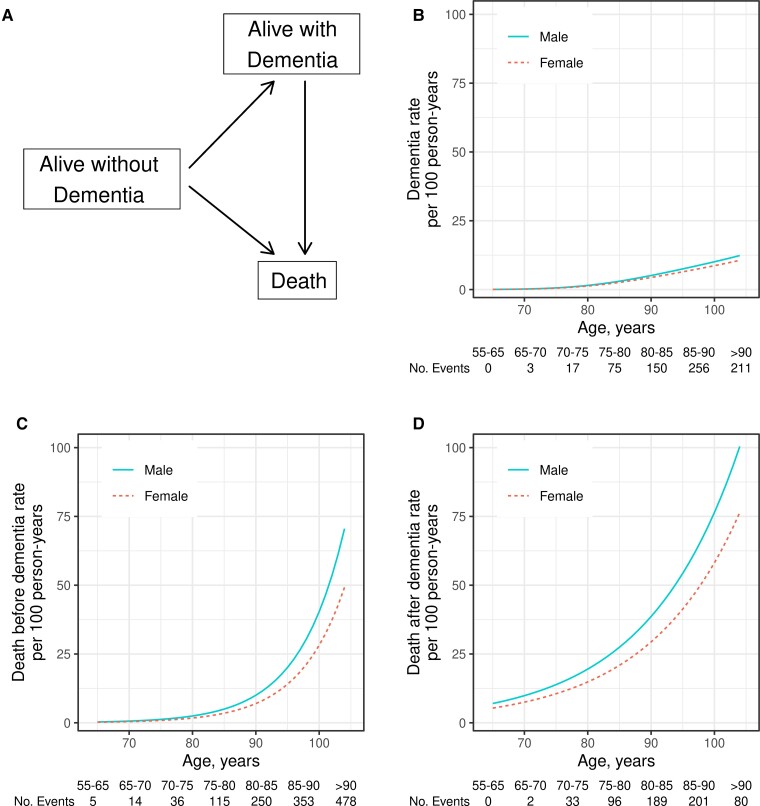
**Multi-state transition model and numbers of mortality and incident dementia events per 100 person-years by sex and age group.** (**A**) Multi-state transition model. The three states in this model are denoted by boxes: alive without dementia, alive with dementia and deceased. The three possible transition paths are denoted by arrows: progression from without dementia to dementia, from without dementia to death and from dementia to death. (**B–D**) Estimated event rates for each transition type by continuous-age separately for men and women; (**B**) incident dementia events per 100 person-years by sex and age, (**C**) death events without dementia per 100 person-years by sex and age and (**D**) death events with dementia per 100 person-years by sex and age. In the tables below the plots in (**B–D**), we show actual event counts by age bin. In (**B-D**), males are denoted by a solid blue line and females by a dashed red line. Estimates are based on the person-years analysis using Poisson models.

The primary analysis was based on a multi-state intensity model,^42^ illustrated in Fig. 1A. All participants started in the without dementia state, and could undergo transitions directly to dementia or death, or undergo sequential transitions to dementia and then to death during follow-up. We used age as the time scale for modelling which seemed more clinically relevant than time in study (see Supplementary material). The multi-state model is parameterized by the transition rates between each pair of states, the estimated future probability of being in each state at each age based on a set of initial conditions and the remaining lifetime risk of ever experiencing dementia.

#### HRs of mortality and incident dementia associated with predictor variables

Each of the three transition rates (represented by the arrows in Fig. 1A) is represented by a separate intensity model, equivalent to a Cox model focused on the end-point of the arrow; HRs and CIs can be interpreted in the same way as a Cox model. Each of the three models included the predictor variables sex, education, APOE status (ɛ4 carrier versus non-carrier), amyloid PET group (normal, moderate, high) and CMC score. Two-way interactions were examined for each of the three transitions (see Supplementary material). For the without dementia to dementia transition, the model included the two interactions with sex that were significant (APOE × sex and amyloid PET × sex). Therefore, we summarize results in the resulting 12 sex × APOE × amyloid predictor variable groups. HRs and CIs for these variables are reported using the overall study group as the reference, i.e. how each group differs from the overall mean (see Supplementary material). Tests for the comparison of two HR estimates were based on a Wald test.

#### Absolute risk predictions

For an individual without dementia at a given age with certain predictor variables, the fitted rate model also leads to estimates of the probability of being in each of the three states at a future age (i.e. *predicted state curves*) and the probability of ever passing through the dementia state before death (i.e. *remaining lifetime risk*). Tests comparing two predicted state curves were based on the area under the curve with jackknife standard errors. Tests for the comparison of two remaining lifetime risk estimates were based on a jackknife standard error.

#### Data availability

The MCSA makes data available to qualified researchers via an online request form at https://ras-rdrs.mayo.edu/Request/IndexRequest.

## Results

### Demographics

Of the 4984 participants in the study, 4336 (87%) were diagnosed as cognitively unimpaired and 648 (13%) as MCI at enrolment (Table 1). The participants had a median age at enrolment of 75 years [inter-quartile range (IQR) 69–81], median education of 14 years (IQR 12–16) and median CMC score of 2 (IQR 1–3) (Table 1 and Supplementary Table 1A); 2463 (49%) were women, 1342 (27%) were APOE ɛ4 carriers and 1786 (36%) underwent amyloid PET imaging. Age, education and diagnosis are also shown by sex, APOE ɛ4 and amyloid PET level in Supplementary Table 1B. In the subset with amyloid PET, the median age was 5 years younger; and, a slightly smaller proportion was MCI at enrolment (10 versus 15%). This subset was otherwise like those that did not undergo amyloid PET (Table 1). The median follow-up time was 9.4 years (maximum 15.7) from enrolment, and 7.5 years from the first available PET.

**Table 1:** Demographics

	Overall (*N* = 4984)	Subset with amyloid PET (*N* = 1786)	Subset without amyloid PET (*N* = 3198)
**Diagnosis, no. (%)**			
CU	4336 (87%)	1604 (90%)	2732 (85%)
MCI	648 (13%)	182 (10%)	466 (15%)
**Age, years**			
Median (Q1, Q3)	75 (69, 81)	72 (65, 77)	77 (72, 83)
Range	55–92	55–90	55–92
**Sex, no. (%)**			
Male	2521 (51%)	955 (53%)	1566 (49%)
Female	2463 (49%)	831 (47%)	1632 (51%)
**Education, years**			
Median (Q1, Q3)	14 (12, 16)	14 (12, 16)	14 (12, 16)
**APOE ɛ4 genotype, no. (%)**			
Carrier	1342 (27%)	510 (29%)	832 (26%)
Non-carrier	3642 (73%)	1276 (71%)	2366 (74%)
**CMC** ^a^			
Median (Q1, Q3)	2 (1, 3)	2 (1, 3)	2 (1, 3)
Range	0–7	0–7	0–7
**Centiloid group at initial amyloid PET, no. (%)**			
Highly elevated (68+)		241 (13%)	
Moderately elevated (22–68)		350 (20%)	
Normal (<22)		1195 (67%)	
**Total dementia events by years after enrolment, no. (%)**			
0–5	278 (39%)	57 (31%)	221 (42%)
5–10	326 (46%)	95 (51%)	231 (44%)
10+	108 (15%)	34 (18%)	74 (14%)
**Total death events by years after enrolment, no. (%)**			
0–5	493 (27%)	54 (14%)	439 (30%)
5–10	843 (46%)	191 (51%)	652 (44%)
10+	516 (28%)	130 (35%)	386 (26%)
**Follow-up, years** ^b^			
Median (95% CI)	9.4 (8.9, 9.7)	8.2 (8.1, 8.3)	10.4 (10.1, 11.0)

Median (Q1, Q3) refers to the median, first and third quartile.

CU, cognitively unimpaired; MCI, mild cognitive impairment; CMC, cardiovascular/metabolic conditions.

^a^ CMC score is the sum of the presence or absence of seven vascular-health-related conditions. A higher score indicates worse cardiometabolic health.

^b^ Median follow-up was estimated using the reverse Kaplan–Meier (see Supplementary material).

### Overall incident dementia and mortality rates by age and sex

Mortality rates, both with and without dementia, increased exponentially with age and were higher in men than women, 1.31-fold (95% CI 1.12–1.54) for those without dementia and 1.43-fold (95% CI 1.28–1.60) for those with dementia (Fig. 1 and Supplementary Fig. 1). Rates of incident dementia increased exponentially with age and were 16% greater in men than women (95% CI 1.01–1.35).

Of the 712 incident dementia events and the 1852 deaths, 418 (59%) and 1066 (58%), respectively, occurred in participants who had previously discontinued in person study follow-up visits and thus were identified through medical record abstraction. Additionally, 434 (61%) incident dementia events and 1359 (73%) deaths occurred 5 or more years after enrolment.

### Associations between hazards and different predictor variables

**Figure 2:**
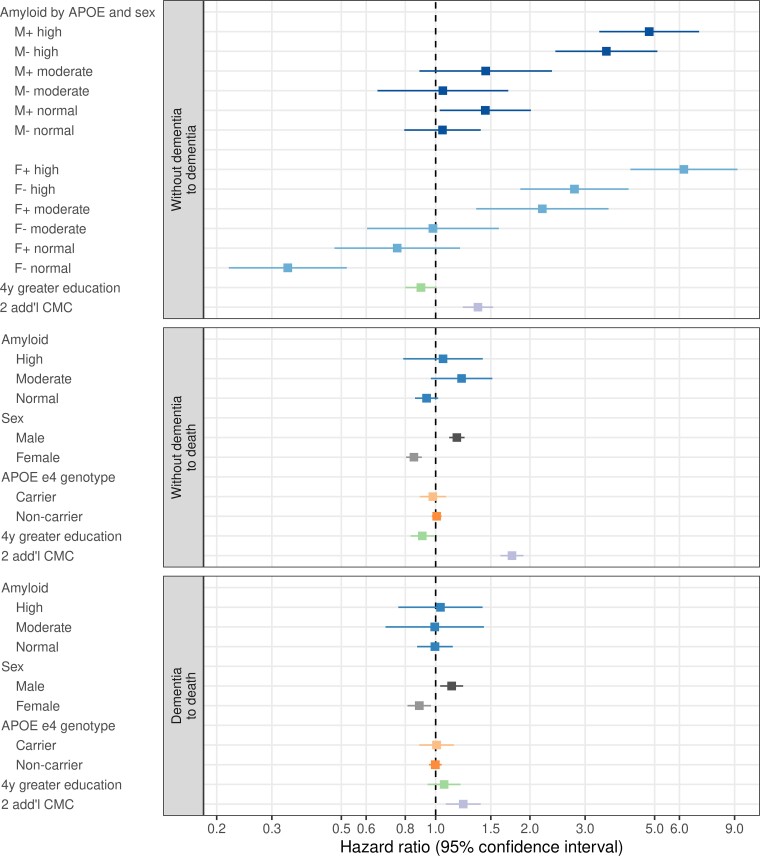
**HRs for state-to-state transitions associated with different subgroups.** Three state-to-state transitions are illustrated: progression from without dementia to dementia, from without dementia to death and from dementia to death. We summarize the association between predictor variables and incidence rates in our multi-state model in the form of HRs where the reference group is the overall study population average for the amyloid, sex and APOE variables. This presentation allows for direct comparison of the rate in a predictor variable subgroup compared to the population average and allows visual comparisons of all possible pairwise comparisons since each HR estimate in the figure shares a common anchor point. The HRs for education and CMC are shown for a specified contrast. + and − symbols in the top panel represent APOE ɛ4 status: + refers to carrier, −refers to non-carrier. HRs estimates are from the multi-state intensity model.

Using the overall study population average as the reference, the HRs associated with different predictor variables are shown in Fig. 2 and Supplementary Table 2 for the three possible state-to-state transitions. Two-way interactions were examined for each of the three transitions and only sex × amyloid and sex × APOE for the without dementia to dementia transition were significant (see Supplementary material).

#### Without dementia to dementia transitions

Among women, the hazard of incident dementia increased monotonically with increasing amyloid level; 2.91 (95% CI 1.37–6.17) for moderate versus normal amyloid and 2.83 (95% CI 1.61–4.99) for high versus moderate amyloid. Among men, the hazards of incident dementia were similar for moderate versus normal amyloid (HR 1.00, 95% CI 0.57–1.76), but were greater for high versus moderate amyloid (HR 3.33, 95% CI 1.92–5.75). Averaging across men, women and APOE, the HR associated with high versus normal amyloid was 2.11 (95% CI 1.43–2.79).

Within each amyloid group, the hazard of incident dementia was higher for APOE ɛ4 carriers than non-carriers for women (HR 2.24, 95% CI 1.80–2.77) and men (HR 1.37, 95% CI 1.09–1.71). The APOE ɛ4 effect was 1.63 (95% CI 1.20–2.23) times greater for women than men, averaged over all three amyloid groups.

Two additional CMC increased the hazard of incident dementia by a factor of 1.37 (95% CI 1.22–1.53). While greater education was mildly protective, the association was not significant.

#### Without dementia to death

The hazard of death among those without dementia was not associated with amyloid level or APOE ɛ4 status but was higher in men than women (HR 1.37, 95% CI 1.22–1.54). More education was slightly protective (HR 0.90, 95% CI 0.83–0.99). Two additional CMC increased the hazard by 1.75 (95% CI 1.61–1.91).

#### Dementia to death

The hazard of death among those with dementia was not associated with amyloid level, APOE ɛ4 status or education but was higher in men than women (HR 1.27, 95% CI 1.07–1.51). Two additional CMC increased the hazard by 1.22 (95% CI 1.08–1.39).

### Absolute risk

**Figure 3:**
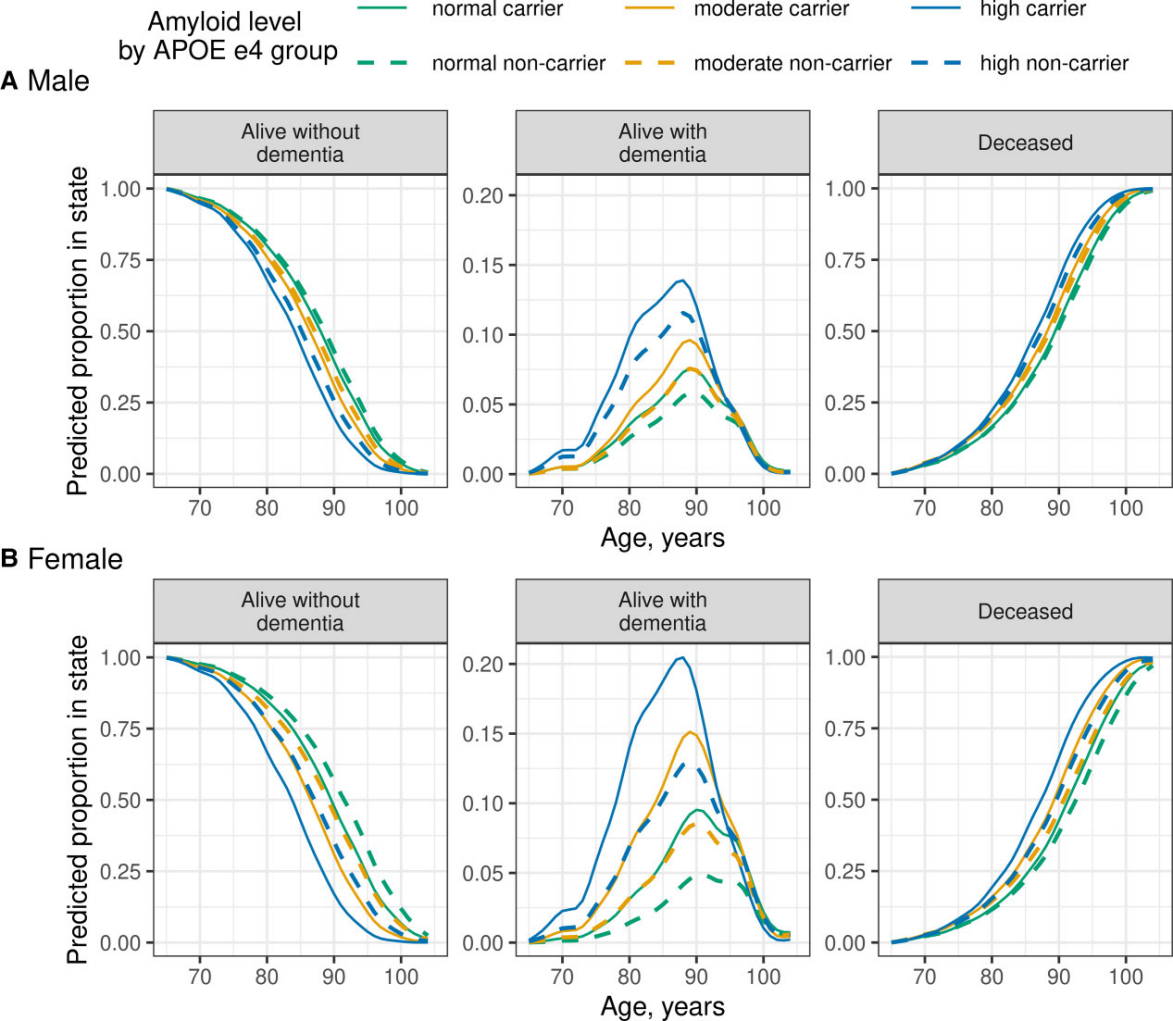
**Absolute predictions by age associated with different predictor variable subgroups.** The two rows illustrate different risk predictor variable subgroups, amyloid PET level by APOE ɛ4 group among (**A**) men and (**B**) women. Other predictor variable effects were weighted to the frequencies observed in the overall study population. Amyloid PET level is indicated by colour (green = normal, gold = moderate, blue = high) while line type indicates APOE e4 group (solid = carrier, dashed = non-carrier). The columns are arranged by the three possible states in the multi-state model illustrated in Fig. 1A: alive without dementia, alive with dementia or deceased. The columns are also arranged from left to right to reflect group-level change over time in a cohort beginning at age 65 years: progression from the alive without dementia category into the alive with dementia or the deceased category. The *y*-axis scale of the middle column (i.e. predicted proportion of original cohort alive with dementia) is limited to a smaller range compared to the other columns to better show the alive with dementia curves. Estimates are based on predicted state curves obtained from the multi-state intensity model.

In Fig. 3, we show predicted state (alive without dementia, alive with dementia or deceased) by age for 12 groups defined by combinations of amyloid PET, sex and APOE based on an exemplar cohort of individuals without dementia at age 65. Figure 3 graphically illustrates associations between different predictor variables and the competing risks of death and dementia. Predicted average time with dementia (equivalent to area under the curve) varied considerably across the 12 predictor variable groups from a maximum of 3.35 (95% CI 2.64–4.06) years in APOE ɛ4 carrier high amyloid women to 0.75 (95% CI 0.51–0.99) years in APOE ɛ4 non-carrier normal amyloid women (Supplementary Table 3).

**Figure 4:**
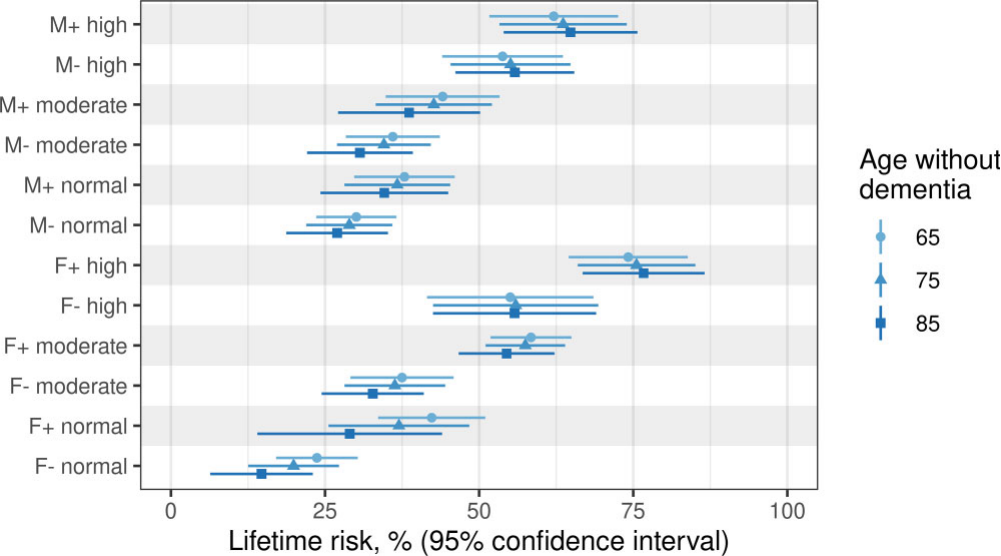
**Remaining lifetime risk of dementia by sex, APOE genotype, and amyloid group for a person without dementia at starting ages 65, 75 or 85 years.** The remaining lifetime risk for all 12 combinations of amyloid group, sex and APOE ɛ4 comes from the competing risks model and is averaged over all the combinations of education and CMC. The standard deviation for the remaining lifetime risk was computed using a grouped jackknife with 20 groups. Estimates are based on remaining lifetime risk estimates obtained from the multi-state intensity model.

Figure 4 illustrates the estimated remaining lifetime risk of experiencing dementia for groups defined by combinations of amyloid PET, sex and APOE who were alive and without dementia at 65, 75 and 85 years. The remaining lifetime risk was constant across different starting ages for high amyloid men and women but declined slightly with advancing age for lower risk groups (Fig. 4 and Supplementary Table 4). Patterns of remaining lifetime risk of dementia varied considerably by APOE/sex/amyloid group (Fig. 4) and mirrored those for HRs (Fig. 2). However, the remaining lifetime risk was greater for women than men in the predictor variable groups most characteristic of Alzheimer’s disease. For example, among APOE ɛ4 carriers with moderate amyloid levels, the remaining lifetime risk of dementia at age 65 years was 58% (95% CI 52–65%) for women compared to 44% (95% CI 35–53%) among men. Among APOE ɛ4 carriers with high levels of amyloid, the risk increased to 74% (95% CI 65–84%) for women compared to 62% (95% CI 52–73%) for men (Supplementary Table 4).

### Sensitivity analyses

We performed sensitivity analyses of HRs and lifetime risk (referenced to Figs 2 and 4, respectively, in the main analyses) in the subset of individuals with amyloid PET. As expected due to larger sample size, the CIs were narrower for non-amyloid PET covariates in the full sample. There were no important differences in either the HRs (Supplementary Fig. 2) or the lifetime risk estimates (Supplementary Fig. 3) when the subset with amyloid PET was compared with the full sample—i.e. CIs overlap considerably for each variable between the two sample sets.

## Discussion

Most Alzheimer’s biomarker studies have focused on relative hazards (i.e. HRs, or relative rates); however, here we also estimated the absolute risk of dementia (Figs 3 and 4). HRs represent the ratio of rates of an event in individuals with a predictor variable pattern relative to a reference group. While informative, they do not provide individuals with probabilistic estimates of the likelihood that they will experience an outcome. In contrast, the remaining lifetime risk reflects how likely an individual with a predictor variable pattern is to experience the event in their remaining lifetime. To our knowledge, only one prior Alzheimer’s biomarker study^9^ has addressed remaining lifetime risk, and none have done so within a defined population.

Figure 3 illustrates the importance of the competing risk of mortality on the absolute risk of dementia. Knowledge of both dementia and death rates is needed to estimate the remaining lifetime risk of dementia.^9,43–45^ Prior studies examining age and sex alone as predictors most often observed that the remaining lifetime risk of incident dementia was constant or declined modestly with increasing age in both sexes.^43–45^ We examined remaining lifetime risk associated with various predictor variables beyond age and sex and found that remaining lifetime risk remained relatively constant across different starting ages for the highest risk groups, particularly those with high amyloid, but declined slightly with older starting age for lower risk groups (Fig. 4 and Supplementary Table 4). A likely explanation is that among persons with high amyloid, older individuals are as likely to experience dementia before death as younger individuals. In contrast, among persons with normal amyloid, older individuals are more likely to die without dementia than younger individuals because younger individuals have greater opportunity to develop abnormal amyloid and dementia in their remaining lifetime.

While absolute risk estimates are more clinically meaningful, it is also useful to compare HRs (i.e. relative rates) from our study with prior epidemiological literature. Therefore, we first estimated overall incident dementia and death rates by age and sex alone. Reported dementia incidence rates vary^46,47^; however, consistently reported findings include exponential increases in mortality and dementia rates with age, and higher mortality rates in men than women.^46–48^ We found slightly greater dementia incidence rates in men than women overall (Fig. 1 and Supplementary Fig. 1) which is consistent with most studies conducted in the USA, but not with all studies from other countries.^10,46,47,49–54^ Consistent with prior literature,^55^ we also found that mortality rates were higher in those with dementia prior to death versus those without dementia.

HRs of incident dementia varied considerably by predictor variable group (Fig. 2 and Supplementary Table 2). Higher amyloid levels and APOE ɛ4 most strongly increased the hazard of incident dementia in women and men. This was anticipated based on prior work showing both higher levels of amyloid PET^7,11,12,17–24,56–61^ and APOE ɛ4^13,24,57,62–69^ are associated with cognitive decline and dementia. More interesting were the complex relationships between sex and both amyloid and APOE ɛ4.

While not replicated universally,^10,14,70^ some studies have identified a stronger association between APOE ɛ4 and dementia or cognitive decline in women than men.^15,27,71^ However, β-amyloid and APOE ɛ4 are closely related to each other. APOE ɛ4 increases the likelihood of and lowers the age of onset of both amyloidosis and dementia.^13,72,73^ To gain more comprehensive understanding, it is necessary to examine the separate sex × amyloid and sex × APOE ɛ4 interactions. Doing so, we found that APOE ɛ4 had a stronger association with incident dementia in women than men across all amyloid levels. At the same time, the combination of APOE ɛ4 non-carrier with low amyloid was selectively more protective in women than men (Fig. 2 and Supplementary Table 2). This set of findings implies different biological effects in women versus men.^15,27,71,74,75^

We also found a sex difference in the relationship between amyloid PET level and the hazard of incident dementia. Among women the relationship was a monotonic increase; in contrast, normal and moderate amyloid men had nearly equal hazards (Fig. 2 and Supplementary Table 2). One possible explanation for different associations by sex may be that a normal amyloid level is not as protective against dementia in men as in women. Dementia in elderly persons is typically not due to Alzheimer’s disease alone but rather due to more than one disease process.^1–3^ Both vascular risk factors^76^ and Lewy body disease^77,78^ are more common in men.

Of the predictor variables examined only male sex, less education and increased CMC were associated with an increased hazard of mortality. Prior studies report mixed conclusions on the association between APOE ɛ4 and mortality.^63,79–81^ It may seem counterintuitive that the two predictor variables in our model that are characteristic of Alzheimer’s disease, APOE ɛ4 and β-amyloid, had no direct association with the hazard of mortality (nor were their interactions with sex significant for mortality) (Fig. 2 and Supplementary Table 2). One possible explanation is that while predictor variables characteristic of Alzheimer’s disease increase the hazard of dementia, once an individual has dementia, mortality rates are not highly dependent on the specific aetiology.

The fact that higher amyloid PET levels increased the hazard, and more importantly, the remaining lifetime risk of incident dementia (Figs 2–4 and Supplementary Tables 2–4) is relevant to current Alzheimer’s disease clinical trials which often target β-amyloid.^82,83^ Monoclonal antibodies that target fibrillar forms of β-amyloid can effectively decrease amyloid load.^84,85^ While our data are observational and therefore cannot prove that removing amyloid would reduce incidence rates or absolute risk of dementia, the results show that those with higher amyloid progress to dementia at faster rates and have significantly higher lifetime risk for dementia than those with normal levels. At present, anti-amyloid clinical trials require an abnormal amyloid biomarker study for inclusion. The fact that hazard and remaining lifetime risk of incident dementia varied dramatically by the subgroups examined suggests that it might be useful to take a more granular approach to inclusion and stratification based on combinations of sex, APOE ɛ4 and amyloid level.

The US Food and Drug Administration recently approved aducanumab for individuals in the MCI or mild dementia phases of Alzheimer’s disease. Most individuals in this study were cognitively unimpaired at baseline and therefore results of this study are only somewhat relevant to current clinical care considerations. Information in this study nonetheless has practical clinical value in life planning for elderly individuals without dementia, value for assessing the utility of combined biomarker and genetic screening of individuals without dementia, and value for assessing the potential public health impact of interventions.

This study has some limitations. Including the entire MCSA cohort over age 55 years rather than only those with amyloid PET studies allowed us to strengthen estimates of the associations between other predictor variables and outcomes as shown in the sensitivity analyses (Supplementary Figs 2 and 3). However, only 36% of participants underwent amyloid PET which is not ideal.

This cohort is from a population-based sample and so by design reflects the *de facto* demographics of Olmsted County, MN, USA of which the majority is non-Hispanic White. Results may differ in populations with different patterns of social determinants of health^86–88^; however, the predictor variables examined in this study do exist in all populations.

Ascertainment of amyloidosis was based on amyloid PET which may not be available in some settings. However, recent reports indicate high correlation between amyloid PET and plasma biomarkers.^89–91^ Future work should assess if similar associations are found between plasma biomarkers and the absolute risk of dementia reported here with amyloid PET.

## Supplementary Material

fcac017_Supplementary_Data

## Abbreviations

CMC =: cardiovascular/metabolic conditions
CI =: confidence interval
HR =: hazard ratio
MCSA =: Mayo Clinic Study of Aging
MCI =: mild cognitive impairment
SUVR =: standardized uptake value ratio
