# Supplementary material for: Long-term associations between amyloid positron emission tomography, sex, apolipoprotein E and incident dementia and mortality among individuals without dementia: hazard ratios and absolute risk
Source: Brain Commun. 2022 Feb 2;4(2):fcac017. doi: 10.1093/braincomms/fcac017 (PMC8924651; doi:10.1093/braincomms/fcac017)
Supplement: fcac017_Supplementary_Data [file fcac017_supplementary_data.docx]

**ONLINE SUPPLEMENTARY MATERIAL INDEX**

**Supplementary figures and tables**

**Supplementary Table 1a. Detailed breakdown of cardiovascular/metabolic (CMC) scores and race**

**Supplementary Table 1b. Demographics by predictor variable group broken out by sex, APOE ε4, and amyloid PET level**

**Supplementary Figure 1. Rates of mortality (without and with dementia) and rates of incident dementia per 100 person-years by age and sex plotted on log scale. (Companion to Figs 1B–D in main text).**

**Supplementary Table 2a. Hazard ratio estimates and 95% confidence intervals (CI) by predictor variable sub group referenced to the overall study population average (companion to Figure 2 in main text)**

**Supplementary Table 2b. P-values for pairwise comparisons of the hazard ratio estimates for the top 12 rows of Supplementary Table 2a and Figure 2 (i.e. without dementia to dementia estimates by sex [M/F], APOE ε4 genotype [–/+], and amyloid PET group [normal/moderate/high]).**

**Supplementary Figure2. Sensitivity analysis. Hazard ratios for state to state transitions associated with different predictor variable groups. Comparing HRs from the model fit among the overall sample (solid squares) against HRs from the model fit among the subset having amyloid-PET imaging (open circles).**

**Supplementary Table 3a. Area under the predicted probability of being in the “alive with dementia” state at a future age curves (i.e., estimated time in the dementia state) by sex (M/F), APOE ε4 genotype (–/+), and amyloid PET group (normal/moderate/high) for a person without dementia at age 65 years. Areas were calculated between ages 65 and 100. Companion to Figure 3 (middle column) in main text.**

**Supplementary Table 3b. P-values for pairwise comparisons of area under the predicted probability of being in the “alive with dementia” state (i.e., estimated time in the dementia state) shown in Supplementary Table 3a. Areas were calculated between ages 65 and 100 by sex (M/F), APOE ε4 genotype (–/+), and amyloid PET group (normal/moderate/high) for a person without dementia at age 65.**

**Supplementary Table 4a. Remaining lifetime risk of dementia by sex (M/F), APOE ε4 genotype (–/+), and amyloid PET group (normal/moderate/high) for a person without dementia at different starting ages (companion to Figure 4 in text). Values in each cell represent the proportion (%), with 95% confidence interval, of each predictor variable subgroup expected to experience incident dementia during their lifetime.**

**Supplementary Table 4b. P-values for pairwise comparisons of remaining lifetime risk of dementia shown in Supplementary Table 4a. Remaining lifetime risk of dementia was calculated by sex (M/F), APOE ε4 genotype (–/+), and amyloid PET group (normal/moderate/high) for a person without dementia at age 65 years.**

**Supplementary Figure 3. Sensitivity analysis. Lifetime risk of dementia by sex, APOE genotype, and amyloid group for those non-demented at ages 65 through 85 by 10-year increments. Comparison estimates from the model fit among the full sample to estimates from the model fit among the subset with amyloid PET.**

**Additional description of Mayo Clinic Study on Aging**

**Statistical supplement**

**Supplementary Figure 4. Hazard ratios for without dementia to dementia, without dementia to death, and dementia to death transitions by age for CMC.**

**Supplementary Figure 5. Hazard ratios for the without dementia to dementia transition by age for the APOE and sex predictor variable subgroups.**

**Supplementary Figure 6. Comparison of the predicted proportion in the alive with dementia state by age between the age constant model (main model, dashed lines) vs the model with age varying hazards for CMC and APOE (age-dependent coefficients, solid lines).**

**Supplementary Figure 7. Comparison of the remaining lifetime risk of ever experiencing dementia between the age constant model (main model, dashed lines) vs the model with age varying hazards for CMC and APOE (age-dependent coefficients, solid lines).**

**Supplementary figures and tables**

**Supplementary Table 1a. Detailed breakdown of cardiovascular/metabolic (CMC) scores**

|  | **Overall (N=4984)** | **Subset  with  amyloid-PET (N=1786)** | **Subset  without amyloid-PET (N=3198)** |
| --- | --- | --- | --- |
| **CMC, N (%)** |  |  |  |
| 0 | 417 (8%) | 189 (11%) | 228 (7%) |
| 1 | 1085 (22%) | 478 (27%) | 607 (19%) |
| 2 | 1447 (29%) | 544 (30%) | 903 (28%) |
| 3 | 1081 (22%) | 343 (19%) | 738 (23%) |
| 4 | 601 (12%) | 170 (10%) | 431 (13%) |
| 5 | 281 (6%) | 50 (3%) | 231 (7%) |
| 6 | 67 (1%) | 11 (1%) | 56 (2%) |
| 7 | 5 (0%) | 1 (0%) | 4 (0%) |
| *higher CMC values indicate worse cardiometabolic health |  |  |  |
|  |  |  |  |
|  |  |  |  |
|  |  |  |  |
|  |  |  |  |
|  |  |  |  |
|  |  |  |  |

**Supplementary Table 1b. Demographics by predictor variable group broken out by sex, APOE ε4, and amyloid PET level**

|  | Female ε4− Normal Amyloid (N=432) | Female ε4+ Normal Amyloid (N=118) | Female ε4− Moderate Amyloid (N=103) | Female ε4+ Moderate Amyloid (N=66) | Female ε4− High Amyloid (N=46) | Female ε4+ High Amyloid (N=66) | Male ε4− Normal Amyloid (N=518) | Male ε4+ Normal Amyloid (N=127) | Male ε4− Moderate Amyloid (N=117) | Male ε4+ Moderate Amyloid (N=64) | Male ε4− High Amyloid (N=60) | Male ε4+ High Amyloid (N=69) |
| --- | --- | --- | --- | --- | --- | --- | --- | --- | --- | --- | --- | --- |
| **Diagnosis, No. (%)** |  |  |  |  |  |  |  |  |  |  |  |  |
| CU | 404 (94%) | 110 (93%) | 92 (89%) | 60 (91%) | 38 (83%) | 49 (74%) | 471 (91%) | 119 (94%) | 104 (89%) | 55 (86%) | 44 (73%) | 58 (84%) |
| MCI | 28 (6%) | 8 (7%) | 11 (11%) | 6 (9%) | 8 (17%) | 17 (26%) | 47 (9%) | 8 (6%) | 13 (11%) | 9 (14%) | 16 (27%) | 11 (16%) |
| **Age, years** |  |  |  |  |  |  |  |  |  |  |  |  |
| Median (Q1,Q3) | 70 (63, 75) | 68 (62, 73) | 75 (71, 79) | 71 (66, 75) | 79 (74, 83) | 74 (72, 78) | 71 (64, 76) | 67 (61, 72) | 76 (72, 80) | 75 (71, 81) | 79 (75, 83) | 76 (73, 82) |
| Range | 55 - 90 | 55 - 88 | 56 - 90 | 59 - 85 | 64 - 90 | 62 - 89 | 55 - 89 | 55 - 86 | 55 - 88 | 56 - 90 | 67 - 90 | 67 - 90 |
| **Education, years** |  |  |  |  |  |  |  |  |  |  |  |  |
| Median (Q1,Q3) | 14 (12, 16) | 14 (12, 16) | 14 (12, 16) | 13 (12, 16) | 14 (12, 16) | 14 (12, 16) | 15 (12, 18) | 16 (12, 16) | 14 (12, 17) | 16 (12, 17) | 16 (13, 18) | 16 (12, 18) |
| Range | 0 - 20 | 8 - 20 | 8 - 20 | 12 - 19 | 8 - 20 | 8 - 20 | 6 - 20 | 7 - 20 | 6 - 20 | 8 - 20 | 8 - 20 | 8 - 20 |
| **Ethnicity, No. (%)** |  |  |  |  |  |  |  |  |  |  |  |  |
| Not hispanic | 428 (99%) | 118 (100%) | 101 (99%) | 66 (100%) | 46 (100%) | 65 (100%) | 514 (99%) | 125 (100%) | 116 (100%) | 64 (100%) | 59 (100%) | 69 (100%) |
| Hispanic | 3 (1%) | 0 | 1 (1%) | 0 | 0 | 0 | 1(1%) | 0 | 0 | 0 | 0 | 0 |
| **Race, No. (%)** |  |  |  |  |  |  |  |  |  |  |  |  |
| Asian | 1 (1%) | 1 (1%) | 0 | 0 | 0 | 1 (2%) | 3 (1%) | 0 | 0 | 0 | 0 | 2 (3%) |
| Black or African American | 2 (1%) | 1 (1%) | 0 | 0 | 0 | 0 | 2 (1%) | 0 | 0 | 0 | 0 | 0 |
| White | 425 (99%) | 116 (98%) | 99 (97%) | 66 (100%) | 46 (100%) | 65 (98%) | 507 (98%) | 126 (100%) | 115 (99%) | 63 (98%) | 59 (100%) | 66 (96%) |
| More than one | 3 (1%) | 0 | 3 (3%) | 0 | 0 | 0 | 4 (1%) | 0 | 1 (1%) | 1 (2%) | 0 | 1 (1%) |


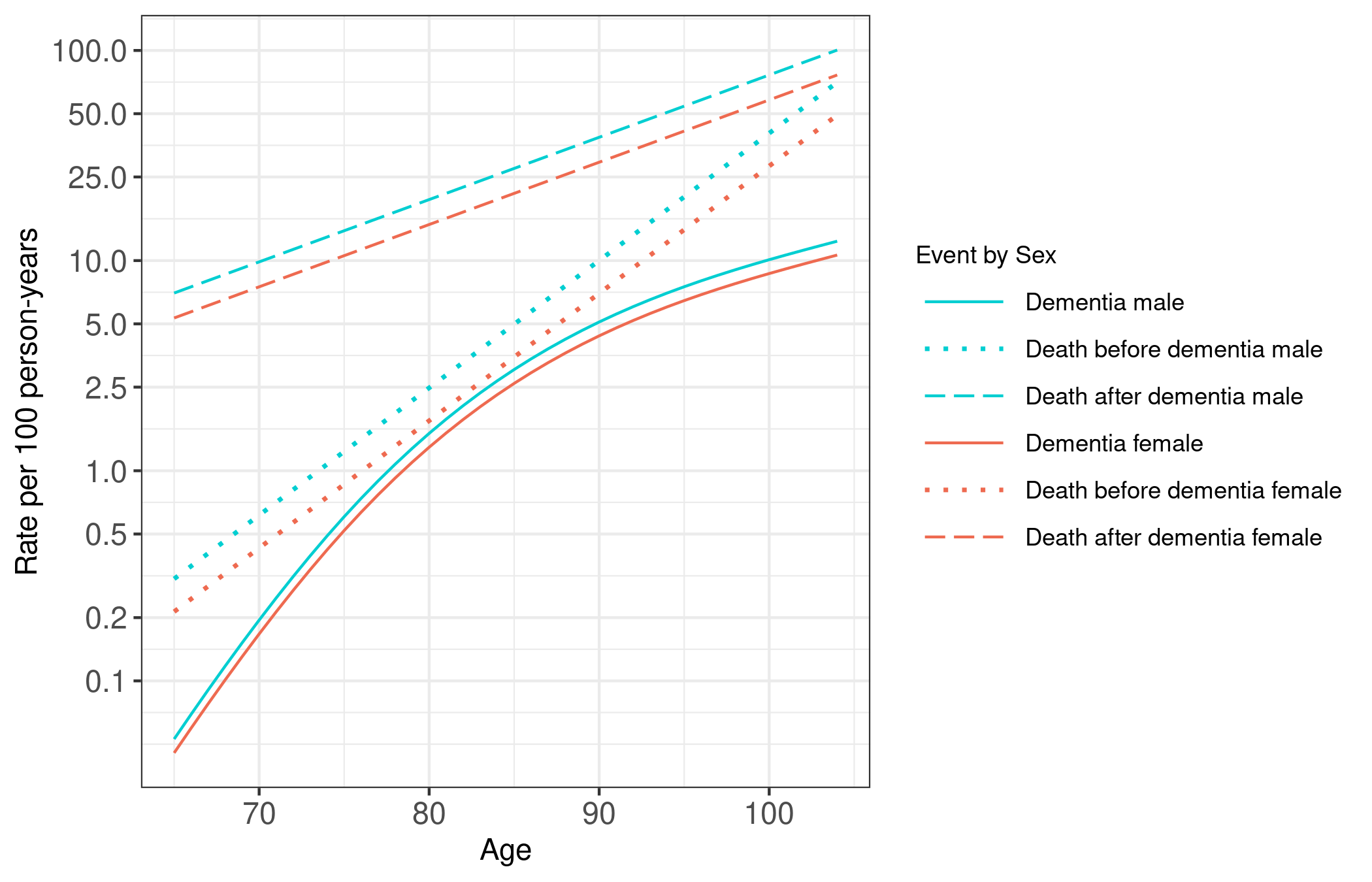


**Supplementary Figure 1. Rates of mortality (without and with dementia) and rates of incident dementia per 100 person-years by age and sex plotted on log scale. (Companion to Figs 1B–D in main text).** When plotted on log scale, the major findings are more easily visualized: death rates without dementia increased 4-fold every 10 years (95% CI 3.70–4.40)**,** rates of death among those with dementia increased 2-fold every 10 years (95% CI 1.70–2.31), and incident dementia rates increased exponentially to about age 90 years after which the rate of increase slowed. Mortality rates without or with dementia were 1.43 (95% CI 1.28–1.60) and 1.31 (95% CI 1.12–1.54) fold higher in men than women, respectively. Rates of incident dementia were 1.16 fold higher in men than women (95% CI 1.01–1.35).

**Supplementary Table 2a. Hazard ratio estimates and 95% confidence intervals (CI) by predictor variable sub group referenced to the overall study population average (companion to Figure 2 in main text)**

| **Transition** | **Variable** | **HR** | **95% CI** |
| --- | --- | --- | --- |
| **Without dementia to dementia** |  |  |  |
|  | Male ε4+ high amyloid | 4.80 | (3.33, 6.94) |
|  | Male ε4− high amyloid | 3.51 | (2.41, 5.10) |
|  | Male ε4+ moderate amyloid | 1.44 | (0.89, 2.35) |
|  | Male ε4− moderate amyloid | 1.05 | (0.65, 1.71) |
|  | Male ε4+ normal amyloid | 1.44 | (1.03, 2.01) |
|  | Male ε4− normal amyloid | 1.05 | (0.79, 1.39) |
|  |  |  |  |
|  | Female ε4+ high amyloid | 6.20 | (4.18, 9.19) |
|  | Female ε4− high amyloid | 2.77 | (1.86, 4.13) |
|  | Female ε4+ moderate amyloid | 2.19 | (1.35, 3.56) |
|  | Female ε4− moderate amyloid | 0.98 | (0.60, 1.59) |
|  | Female ε4+ normal amyloid | 0.75 | (0.48, 1.20) |
|  | Female ε4− normal amyloid | 0.34 | (0.22, 0.52) |
|  |  |  |  |
|  | 4y greater education | 0.90 | (0.80, 1.01) |
|  | 2 additional CMC | 1.37 | (1.22, 1.53) |
| **Without dementia to death** |  |  |  |
|  | High amyloid | 1.06 | (0.79, 1.41) |
|  | Moderate amyloid | 1.21 | (0.96, 1.52) |
|  | Normal amyloid | 0.94 | (0.86, 1.02) |
|  | Male | 1.17 | (1.10, 1.24) |
|  | Female | 0.85 | (0.80, 0.90) |
|  | APOE ε4 carrier | 0.98 | (0.89, 1.08) |
|  | APOE ε4 non-carrier | 1.01 | (0.97, 1.04) |
|  | 4y greater education | 0.91 | (0.83, 0.99) |
|  | 2 additional CMC | 1.75 | (1.61, 1.91) |
| **Dementia to death** |  |  |  |
|  | High amyloid | 1.04 | (0.76, 1.41) |
|  | Moderate amyloid | 0.99 | (0.69, 1.43) |
|  | Normal amyloid | 0.99 | (0.87, 1.13) |
|  | Male | 1.12 | (1.03, 1.22) |
|  | Female | 0.89 | (0.81, 0.97) |
|  | APOE ε4 carrier | 1.01 | (0.89, 1.14) |
|  | APOE ε4 non-carrier | 1.00 | (0.95, 1.05) |
|  | 4y greater education | 1.06 | (0.94, 1.20) |
|  | 2 add’l CMC | 1.22 | (1.08, 1.39) |

**Supplementary Table 2b. P-values for pairwise comparisons of the hazard ratio estimates for the top 12 rows of Supplementary Table 2a and Figure 2 (i.e. without dementia to dementia estimates by sex [M/F], APOE ε4 genotype [–/+], and amyloid PET group [normal/moderate/high]).**

|  | **F− normal** | **M− normal** | **F+ normal** | **M+ normal** | **F− moderate** | **M− moderate** | **F+ moderate** | **M+ moderate** | **F−  high** | **M− high** | **F+ high** |
| --- | --- | --- | --- | --- | --- | --- | --- | --- | --- | --- | --- |
| M− normal | <0.001 |  |  |  |  |  |  |  |  |  |  |
| F+ normal | <0.001 | 0.34 |  |  |  |  |  |  |  |  |  |
| M+ normal | <0.001 | 0.006 | 0.07 |  |  |  |  |  |  |  |  |
| F− moderate | 0.005 | 0.81 | 0.52 | 0.21 |  |  |  |  |  |  |  |
| M− moderate | 0.003 | >0.99 | 0.40 | 0.33 | 0.83 |  |  |  |  |  |  |
| F+ moderate | <0.001 | 0.01 | 0.005 | 0.17 | <0.001 | 0.03 |  |  |  |  |  |
| M+ moderate | <0.001 | 0.28 | 0.10 | >0.99 | 0.26 | 0.006 | 0.23 |  |  |  |  |
| F− high | <0.001 | <0.001 | <0.001 | 0.01 | <0.001 | 0.001 | 0.45 | 0.03 |  |  |  |
| M− high | <0.001 | <0.001 | <0.001 | <0.001 | <0.001 | <0.001 | 0.11 | 0.004 | 0.33 |  |  |
| F+ high | <0.001 | <0.001 | <0.001 | <0.001 | <0.001 | <0.001 | <0.001 | <0.001 | <0.001 | 0.02 |  |
| M+ high | <0.001 | <0.001 | <0.001 | <0.001 | <0.001 | <0.001 | 0.007 | <0.001 | 0.02 | 0.006 | 0.28 |


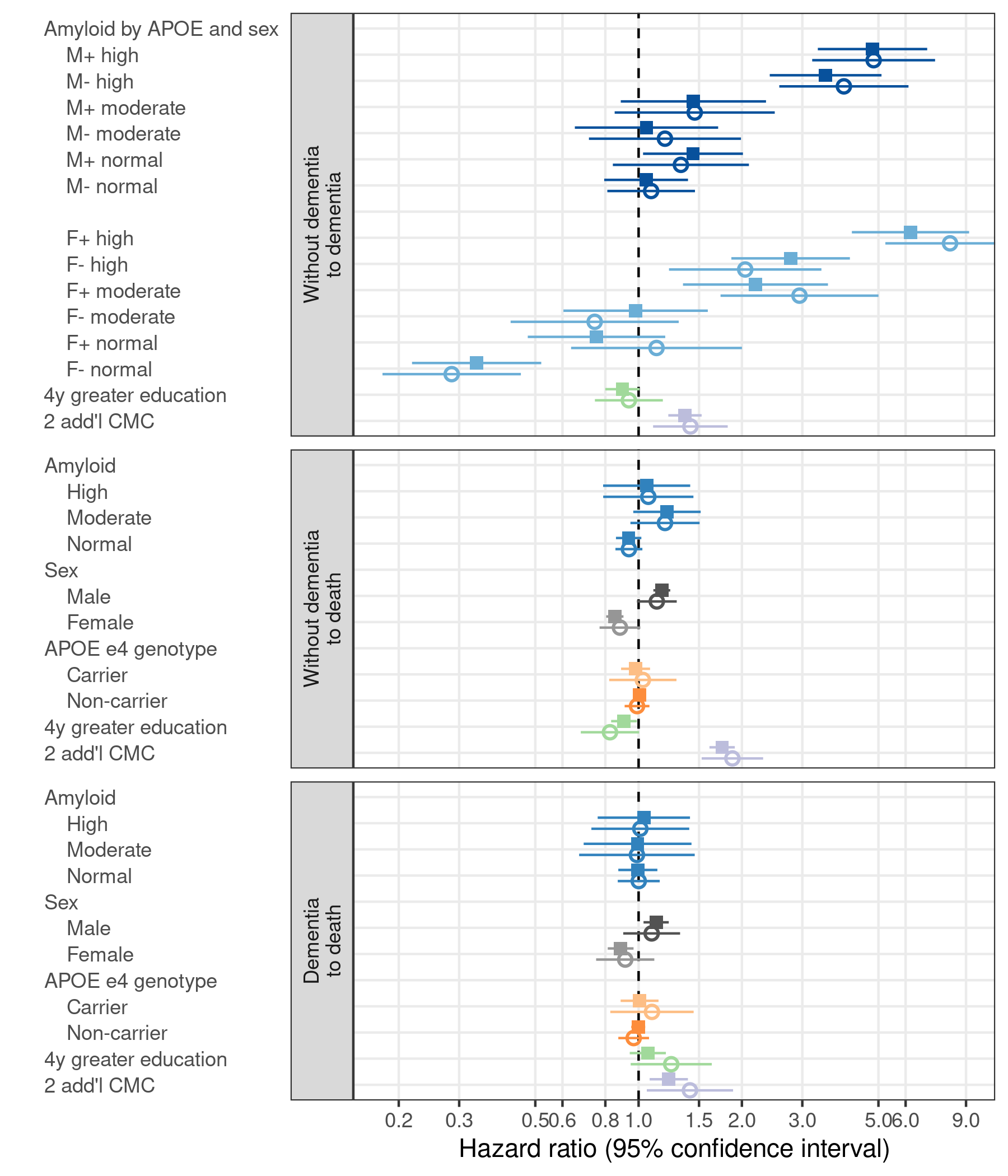


**Supplementary Figure2. Sensitivity analysis. Hazard ratios for state to state transitions associated with different predictor variable groups. Comparing HRs from the model fit among the overall sample (solid squares) against HRs from the model fit among the subset having amyloid-PET imaging (open circles).**

**Supplementary Table 3a. Area under the predicted probability of being in the “alive with dementia” state at a future age curves (i.e., estimated time in the dementia state) by sex (M/F), APOE ε4 genotype (–/+), and amyloid PET group (normal/moderate/high) for a person without dementia at age 65 years. Areas were calculated between ages 65 and 100. Companion to Figure 3 (middle column) in main text.**

| **Group** | **Alive with Dementia** |
| --- | --- |
| M+ high amyloid | 2.27 (1.59, 2.95) |
| M− high amyloid | 1.88 (1.35, 2.4) |
| M+ moderate amyloid | 1.42 (1.02, 1.83) |
| M− moderate amyloid | 1.13 (0.86, 1.41) |
| M+ normal amyloid | 1.18 (0.65, 1.72) |
| M− normal amyloid | 0.92 (0.52, 1.31) |
|  |  |
| F+ high amyloid | 3.35 (2.64, 4.06) |
| F− high amyloid | 2.13 (1.43, 2.84) |
| F+ moderate amyloid | 2.3 (1.84, 2.76) |
| F− moderate amyloid | 1.32 (0.97, 1.67) |
| F+ normal amyloid | 1.44 (0.98, 1.89) |
| F− normal amyloid | 0.75 (0.51, 0.99) |

**Supplementary Table 3b. P-values for pairwise comparisons of area under the predicted probability of being in the “alive with dementia” state (i.e., estimated time in the dementia state) shown in Supplementary Table 3a. Areas were calculated between ages 65 and 100 by sex (M/F), APOE ε4 genotype (–/+), and amyloid PET group (normal/moderate/high) for a person without dementia at age 65.**

|  | **F− normal** | **M− normal** | **F+ normal** | **M+ normal** | **F− moderate** | **M− moderate** | **F+ moderate** | **M+ moderate** | **F− high** | **M− high** | **F+ high** |
| --- | --- | --- | --- | --- | --- | --- | --- | --- | --- | --- | --- |
| M− normal | 0.42 |  |  |  |  |  |  |  |  |  |  |
| F+ normal | <0.001 | 0.044 |  |  |  |  |  |  |  |  |  |
| M+ normal | 0.13 | 0.07 | 0.35 |  |  |  |  |  |  |  |  |
| F− moderate | <0.001 | 0.13 | 0.69 | 0.69 |  |  |  |  |  |  |  |
| M− moderate | 0.04 | 0.23 | 0.27 | 0.84 | 0.29 |  |  |  |  |  |  |
| F+ moderate | <0.001 | <0.001 | <0.001 | <0.001 | <0.001 | <0.001 |  |  |  |  |  |
| M+ moderate | 0.008 | 0.04 | 0.97 | 0.28 | 0.70 | 0.07 | <0.001 |  |  |  |  |
| F− high | <0.001 | 0.006 | 0.17 | 0.07 | 0.007 | 0.003 | 0.69 | 0.09 |  |  |  |
| M− high | <0.001 | 0.001 | 0.26 | 0.055 | 0.06 | <0.001 | 0.23 | 0.09 | 0.39 |  |  |
| F+ high | <0.001 | <0.001 | <0.001 | <0.001 | <0.001 | <0.001 | 0.006 | <0.001 | <0.001 | <0.001 |  |
| M+ high | <0.001 | <0.001 | 0.045 | 0.002 | 0.02 | <0.001 | 0.92 | <0.001 | 0.76 | 0.10 | <0.001 |

**Supplementary Table 4a. Remaining lifetime risk of dementia by sex (M/F), APOE ε4 genotype (–/+), and amyloid PET group (normal/moderate/high) for a person without dementia at different starting ages (companion to Figure 4 in text). Values in each cell represent the proportion (%), with 95% confidence interval, of each predictor variable subgroup expected to experience incident dementia during their lifetime.**

| **Group** | **Age 65** | **Age 70** | **Age 75** | **Age 80** | **Age 85** | **Age 90** |
| --- | --- | --- | --- | --- | --- | --- |
| M+ high amyloid | 62 (52, 73) | 63 (52, 74) | 64 (53, 74) | 64 (53, 74) | 65 (54, 76) | 60 (47, 72) |
| M− high amyloid | 54 (44, 64) | 55 (44, 65) | 55 (45, 65) | 55 (46, 65) | 56 (46, 65) | 51 (40, 62) |
| M+ moderate amyloid | 44 (35, 53) | 44 (35, 54) | 43 (33, 52) | 41 (31, 52) | 39 (27, 50) | 29 (16, 41) |
| M− moderate amyloid | 36 (28, 44) | 36 (28, 44) | 35 (27, 42) | 33 (25, 41) | 31 (22, 39) | 22 (13, 31) |
| M+ normal amyloid | 38 (30, 46) | 37 (29, 46) | 37 (28, 45) | 36 (26, 45) | 35 (24, 45) | 30 (19, 41) |
| M− normal amyloid | 30 (24, 37) | 30 (23, 36) | 29 (22, 36) | 28 (21, 36) | 27 (19, 35) | 23 (15, 32) |
|  |  |  |  |  |  |  |
| F+ high amyloid | 74 (65, 84) | 75 (65, 85) | 76 (66, 85) | 76 (66, 85) | 77 (67, 87) | 72 (62, 83) |
| F− high amyloid | 55 (42, 69) | 56 (42, 69) | 56 (43, 69) | 56 (43, 69) | 56 (43, 69) | 51 (38, 64) |
| F+ moderate amyloid | 58 (52, 65) | 59 (52, 66) | 57 (51, 64) | 57 (50, 64) | 54 (47, 62) | 44 (33, 55) |
| F− moderate amyloid | 37 (29, 46) | 38 (29, 46) | 36 (28, 44) | 36 (27, 44) | 33 (24, 41) | 25 (16, 34) |
| F+ normal amyloid | 42 (34, 51) | 40 (31, 50) | 37 (26, 48) | 34 (21, 47) | 29 (14, 44) | 24 (8, 39) |
| F− normal amyloid | 24 (17, 30) | 22 (15, 29) | 20 (13, 27) | 18 (10, 25) | 15 (6, 23) | 12 (4, 20) |

**Supplementary Table 4b. P-values for pairwise comparisons of remaining lifetime risk of dementia shown in Supplementary Table 4a. Remaining lifetime risk of dementia was calculated by sex (M/F), APOE ε4 genotype (–/+), and amyloid PET group (normal/moderate/high) for a person without dementia at age 65 years.**

|  | **F−  normal** | **M− normal** | **F+  normal** | **M+ normal** | **F− moderate** | **M− moderate** | **F+ moderate** | **M+ moderate** | **F−  high** | **M−  high** | **F+  high** |
| --- | --- | --- | --- | --- | --- | --- | --- | --- | --- | --- | --- |
| M− normal | 0.21 |  |  |  |  |  |  |  |  |  |  |
| F+ normal | <0.001 | 0.055 |  |  |  |  |  |  |  |  |  |
| M+ normal | 0.02 | 0.007 | 0.49 |  |  |  |  |  |  |  |  |
| F− moderate | <0.001 | 0.14 | 0.37 | 0.95 |  |  |  |  |  |  |  |
| M− moderate | 0.02 | 0.048 | 0.31 | 0.64 | 0.74 |  |  |  |  |  |  |
| F+ moderate | <0.001 | <0.001 | <0.001 | <0.001 | <0.001 | <0.001 |  |  |  |  |  |
| M+ moderate | <0.001 | 0.002 | 0.78 | 0.06 | 0.26 | 0.009 | 0.002 |  |  |  |  |
| F− high | <0.001 | 0.001 | 0.09 | 0.04 | <0.001 | 0.01 | 0.54 | 0.19 |  |  |  |
| M− high | <0.001 | <0.001 | 0.08 | 0.003 | 0.002 | <0.001 | 0.36 | 0.051 | 0.85 |  |  |
| F+ high | <0.001 | <0.001 | <0.001 | <0.001 | <0.001 | <0.001 | <0.001 | <0.001 | <0.001 | <0.001 |  |
| M+ high | <0.001 | <0.001 | 0.002 | <0.001 | <0.001 | <0.001 | 0.48 | <0.001 | 0.32 | 0.007 | 0.02 |


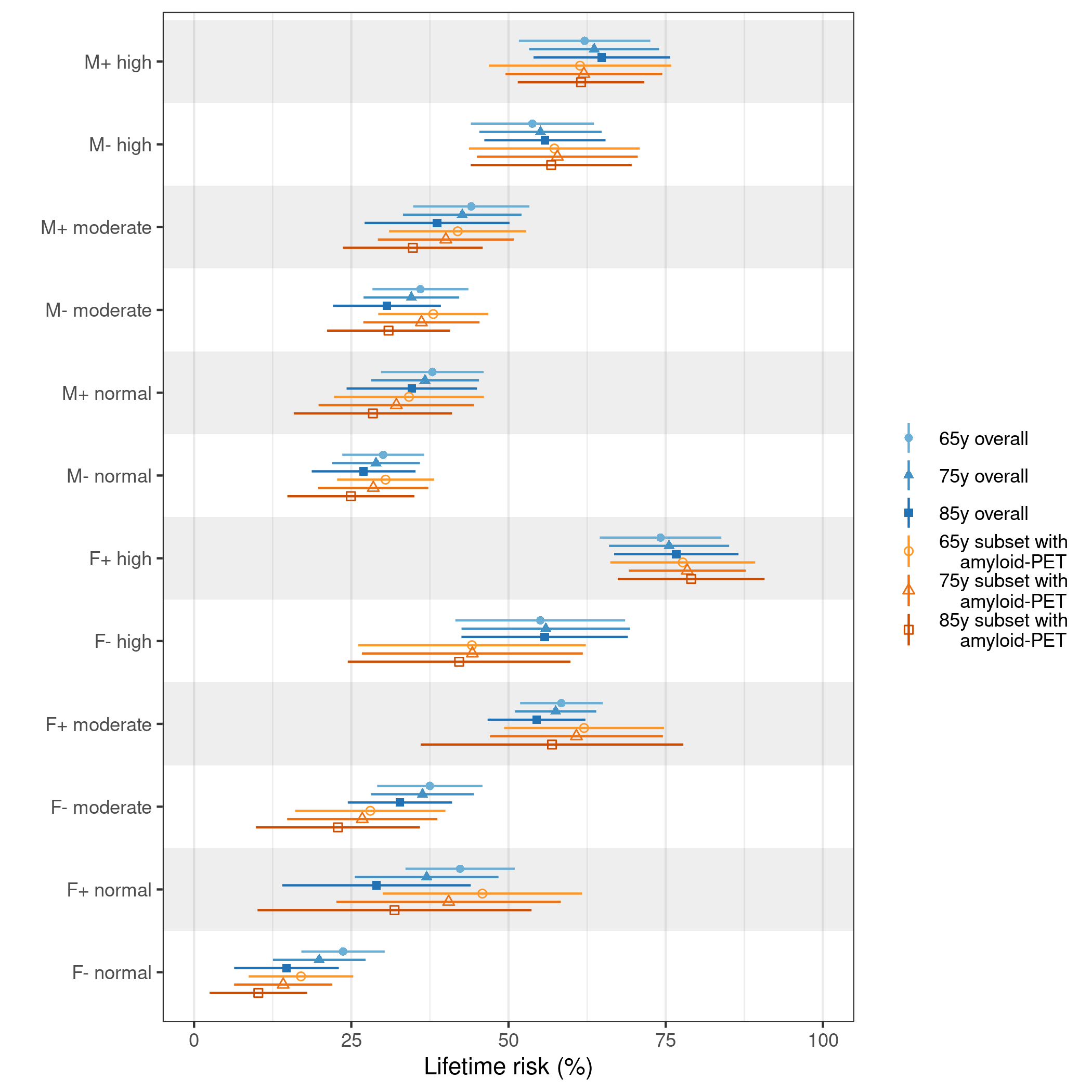


**Supplementary Figure 3. Sensitivity analysis. Lifetime risk of dementia by sex, APOE genotype, and amyloid group for those non-demented at ages 65 through 85 by 10-year increments. Comparison of estimates from the model fit among the full sample to estimates from the model fit among the subset with amyloid PET.** The standard deviation for the lifetime risk was computed using a grouped jack-knife with 20 groups.

**Additional description of Mayo Clinic Study on Aging**

All participants in this study were enrolled in the Mayo Clinic Study of Aging (MCSA) which is a population-based study of cognitive aging among a stratified random sample of a geographically defined population, Olmsted County, Minnesota [1]. Residents aged 30–89 years old were enumerated using the medical records-linkage system of the Rochester Epidemiology Project [2]. From this sampling frame, individuals were randomly selected by 10-year age and sex strata such that men and women were equally represented. Enumeration, stratified random sampling, and screening procedures were repeated to maintain a target of 3,000 active participants who were evaluated approximately every 15 months.

**Follow-up time**

Median follow-up was estimated using the reverse Kaplan-Meier (KM), i.e. a KM of the time to censoring rather than time to event. As pointed out by [3], this is the best estimate of the follow-up experience for a study as a whole (as opposed to the simple median of censoring times, which underestimates survival for the same reason that a median of observed death times underestimates survival).

**Additional description of outcome ascertainment**

Essentially all individuals in Olmsted County receive medical care from either the Mayo Clinic or Olmsted Medical Center. These two healthcare providers participate in the medical records-linkage system of the Rochester Epidemiology Project [2].

Participants in the MCSA are followed from enrollment through all MCSA visits for the events of dementia and death. Death information is identified from the medical record weekly. If a participant withdraws from the MCSA for any reason without having been diagnosed as having dementia during his or her time in the MCSA, the participant’s medical record is reviewed semiannually for a diagnosis of dementia. The entire medical record is reviewed through the current date. Participants that are still alive at the last medical record review and had not received a diagnosis of dementia are re-reviewed until they either have a diagnosis of dementia or are deceased. Medical records are reviewed by nurse abstractors who have extensive experience in medical record review. The date of dementia onset is defined as the first date on which symptoms compatible with a diagnosis of dementia were recorded in the medical record. Prior work has demonstrated that accurate rates of dementia detection can be obtained by passive surveillance using a medical records-linkage system [4].

All participants in this study were without dementia (i.e. had either mild cognitive impairment [MCI] or were cognitively unimpaired) at enrollment. Our primary outcomes were death and dementia. We did not attempt to include MCI as a separate state in our multi state model because we do not believe that MCI can be reliably identified from review of our medical record system.

**Additional description of the CMC variable**

A composite cardiovascular and metabolic conditions (CMC) score was computed for each participant as the sum of seven conditions proposed by the U.S. Department of Health and Human Services as indicators of vascular health: hypertension, hyperlipidemia, cardiac arrhythmias, coronary artery disease, congestive heart failure, diabetes mellitus, and stroke [5, 6]. Nurse-abstracted medical record data were used to identify these seven conditions.

**Choice of vascular disease variable**

Measures of white matter hyperintensity volume from MRI would be a logical variable to add to the analysis. However, in 2017 the MCSA cohort was moved from a GE scanner using a 2D FLAIR acquisition to a Siemens scanner using a higher resolution 3D FLAIR acquisition. This resulted in changes in contrast properties, signal to noise and spatial resolution. We are in the process of developing image processing methods that bridge this data discontinuity, but these developments have not yet been completed or validated.

**Additional description of amyloid PET quantification methods**

Amyloid PET standardized uptake value ratios (SUVR) were formed by normalizing a target region, composed of multiple regions of interest (ROI), to the cerebellar crus gray matter [7]. The amyloid PET target meta-ROI included the prefrontal, orbitofrontal, parietal, temporal, anterior and posterior cingulate, and precuneus ROIs [7]. SUVR values were converted to the Centiloid scale [8, 9].

**Statistical supplement**

**Additional description of the multi-state model:** The multi-state model is shown in **Figure 1a** in the main manuscript. The transition rates (arrows) were fit using the proportional intensity model as described in Putter et al [10]. That is, each of the three transitions can be thought of as a separate Cox model, and describes covariate effects on the rate of that transition. Absolute risk estimates were then calculated from the fitted rate model. Given a starting age in a person without dementia, absolute risk includes the probability of being in each of the three states at a future age (i.e. predicted state curves) and the probability of ever visiting the dementia state (remaining lifetime risk). The area under the predicted state curves estimates the estimated mean number of years spent in each state. For any given starting age/state combination, the expected future trajectory of the individual is represented by probability in state curves, i.e., p(t) = [p(alive and without dementia at time t), p(alive and with dementia at time t), p(deceased at time t)], a vector of 3 values that sum to 1. The curves were computed on discrete time scale, i.e., single years of age, as a product of transition matrices T(a) = exp(R(a)), where R is the rate matrix whose j,k element is the estimated transition rate from state j to state k at that age, estimated from the Cox models, and exp is the matrix exponential. Standard errors were computed using a 20-fold jackknife, refitting the entire model at each iteration. To compare any two probability-in-dementia-state curves we used the area under the curve (AUC) as a simple summary rather than the value at a single age, again with jackknife standard errors. As a footnote, the AUC is also an estimate of the expected cumulative time in the state, a measure that is useful on its own for certain questions, but that interpretation was not central to our use. Computations were done using version 3.2-7 of the survival package in R.

All participants who entered the MCSA study without dementia were used in the analysis, following each until death or last follow-up; 657 without dementia to dementia, 1203 without dementia to death, and 540 dementia to death transitions were observed.

C**hoice of time scale:** The two obvious options for the choice of time scale are to use “time since enrollment” as the underlying time scale and age as an adjusting covariate, or use participant age as the underlying time scale with time since enrollment as a covariate. We chose the latter, as it results in absolute risk estimates that are displayed on an age scale. Absolute risks as a function of age were deemed more relevant to long term health outlook and life planning for elderly individuals than risk as a function of years post enrollment in a study. One consequence of using age as the time scale is that a coefficient and p-value for the effect of age is not produced, but the importance of age as a predictor of both dementia and death is so well established that we felt the benefits of using age as the time scale outweighed the disadvantages. An advantage of using age scale is that within the model all comparisons involve pairs of individuals that are the same age, fully adjusting for this most powerful predictor of both dementia and death. Another advantage of using age as the underlying scale is that the prediction extent is more than doubled from age 65 to 100 (i.e. 35 years) as opposed to the maximum time from enrollment to outcome events (i.e. 15.7 years).

**Splicing:** As stated above one of the advantages of using age scale is that it allows for long-term predictions of absolute risk, e.g. over 35 years, the span from age 65 to 100 in **Figure 4**. Since no single participant has 35 years of follow-up, this result depends on the ability for a participant recruited at age 85, say, to represent the future that will occur, after 20 years, for someone recruited at age 65. That is, individual follow-up trajectories of mean length 9.4 years can be "spliced" together. Validity of this is heavily reliant on the full population capture of the Rochester Epidemiology Project [2] and on the relative stability of the Olmsted county population. (If for instance a substantial fraction of the 85 year olds had worked at a lead smelter which later closed, their clinical trajectory might not represent the future of a current 65 year old.) One of the most subtle aspects of the modeling is the interaction between this and our use of the first available amyloid PET or CMC score as covariates. That is, when a low amyloid PET 65 year old becomes 85, a set of 85 year olds, all with low amyloid at their recruitment age, will be biased healthy, and the predicted future trajectory will be overly optimistic. To deal with this the transition matrices used in calculation of absolute risk were reweighted to reflect the progression in amyloid status over time in our population sample. The same argument applied for CMC.

**Missing data:** Potential participants for this study who were missing covariates (except amyloid PET, see below) were excluded. This included 9 missing education, 246 missing APOE genotype, and 49 missing CMC. Because of the large size of the study, these few exclusions did not impact the power to determine associations between demographic, clinical, and genetic predictors and outcomes. Furthermore, excluding participants missing these values made the analysis straightforward because every predictor variable (except amyloid PET) was available in all participants.

Only 36% of the MCSA cohort received amyloid PET scans. This was due to a combination of factors. One was scanning capacity limitations. Another is some declined to participate in PET; approximately 70% agreed to participate in PET. Finally, the MCSA started enrolling and following participants 5 years prior to initiation of amyloid PET scanning in our study; therefore the earliest MCSA enrollees did not have had the opportunity for amyloid PET.

Information on repeated measures of PET and CMC is not available after participants have withdrawn from in-person study visits, yet the post in-person time interval contains over half of the incident dementia and death events. To avoid informative censoring for these covariates only the initial PET or CMC values were used in the analysis. PET values were categorized into four groups as normal, moderately elevated, highly elevated, and not available (NA). Some participants with PET received their first PET scan sometime after their first visit, and are assigned to the NA group until the PET scan occurred. Retention of the NA group greatly increased the sample size and precision for other key predictors, for example, sex, education, APOE, and CMC.

**Using population averages as the reference:** For the comparison of relative hazards of dementia across the 12 predictor variable subgroups (defined by the combination of sex, APOE, and amyloid PET levels) we elected to use g-formula estimates to display the results. This in effect compares each group to an overall population value rather than electing one of the 12 groups as a ‘reference’; the overall estimate is a simple mean over all subjects in the sample. For the model's risk score, β_1_x_1_ + β_2_x_2_ + ... (the results in **Figure 2**), this computation reduces to the coefficient β_k_ for any variable k that does not include interactions, i.e., the usual result that is reported. For CMC, adding 8 separate estimates to the plot for CMC=0, CMC=1, ..., CMC=7 — all equally spaced since there are no CMC interactions — is unwieldy; however, so the single CMC coefficient was plotted instead; and likewise for the education effect. For time in state and lifetime risk curves the g-estimate is equivalent to the direct adjusted survival estimate [11].

While there is debate in the statistical community on the best way to summarize models with interactions, the g-estimate methods arising from the recent causal modeling literature have the strongest theoretical support, currently [12]. Of the g-estimate methods, the g-formula has a natural interpretation as a population average which is conceptually useful here and has the further advantage of simple implementation.

**Co-variate interactions:**  The models included sex, amyloid level (normal, moderately elevated, highly elevated), APOE ε4 genotype, CMC, and education. For any variables with a univariate association (p < .05) we also investigated all possible 2-way interactions. This was done for each of the 3 transitions. Because of prominence in the exiting literature, we also examined all pairwise interactions among sex, APOE, and amyloid PET. The only significant interactions were sex × APOE (p=0.002) and sex × amyloid PET (p=0.02), and only for incident dementia rates. These were significant and included in the model. The amyloid PET × APOE interaction on incident dementia rates was not significant (p=0.5) and was not included in the model. We considered selected 3-way interactions, but found that the counts in many subsets were too small for reliable estimation (< 10 events), and so did not pursue this avenue further.

**Non-constant effects of APOE and CMC with age:** Due to the wide age span in the cohort, we investigated the possibility of age-dependent effects. Since analyses were done on the age scale, these appear in the Cox model as non-proportional hazards. Tests for non-proportional hazards were done for all covariates, for all three transitions, using the score test of Grambsch and Therneau [13]. Of all combinations of transitions and covariates examined, non-proportional hazards were met for only four. CMC showed age dependent effects for all three transitions and APOE ε4 for the without dementia to dementia transition; all other p-values were > 0.05. We computed a more formal estimate of the age dependent effect for each of these four using a model with age-dependent coefficients. **Supplementary Figure 4** shows the estimated effects as a function of age for all 3 transitions for CMC, which is essentially a linear decline in effect magnitude with age for all 3 transitions. As a reference, the age-independent estimates of the CMC hazard from **Supplementary Table 2a** are shown as 3 points on the right margin of **Supplementary Figure 4**. Since dementia and death rates rise steeply with age (**Figure 1**), this ‘overall’ CMC effect is strongly weighted towards the effect at higher ages, where most dementia and death events occur. Likewise with the APOE × sex interaction shown in **Supplementary Figure 5**; at younger ages the APOE ε4+ vs ε4− difference for females is much higher than that for males, attenuating over time. But again, the ‘overall’ APOE effect is strongly weighted towards the effect at higher ages, where most incident dementia events occur.

**Supplementary Figures 6** and **7** show that although the age-dependent changes in hazard ratios can be large, the practical effect of these age-dependent effects on the absolute risks are small, both in terms of the predicted future probability of dementia for given starting state and age and the remaining lifetime risk of dementia. A primary reason for this is that the ages with the largest effects on the hazard are also ages at which almost none of the incident dementia events occur (**Figure 1**). A small rate, doubled, is still small. In addition, the use of an age-dependent rate versus use of an age-averaged rate has less effect on a cumulative variable like lifetime risk and lifetime risk depends on both dementia and death rates. While age-varying effects did exist for CMC and APOE, because these had little impact on the absolute risk estimates, we did not include them in the main model.


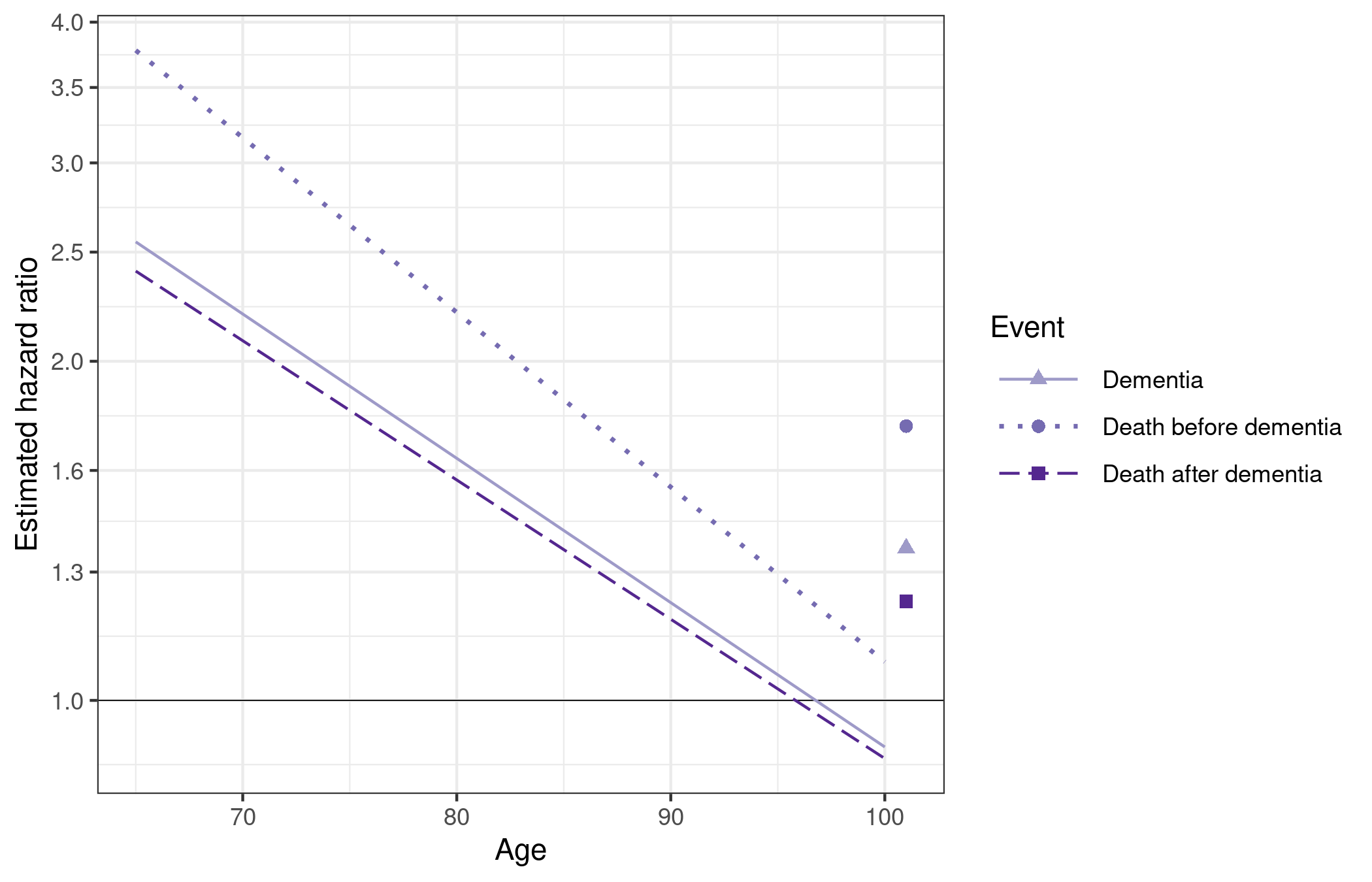


**Supplementary Figure 4. Hazard ratios for without dementia to dementia, without dementia to death, and dementia to death transitions by age for CMC.** The lines represent the estimated hazard ratio for 2 additional CMC conditions at each age from the model with an age-dependent CMC covariate (i.e. age × CMC interaction). As a reference, points at the right side of the figure indicate the hazard ratios from the main model without an age-dependent CMC covariate which are shown in Supplementary Table 2a.


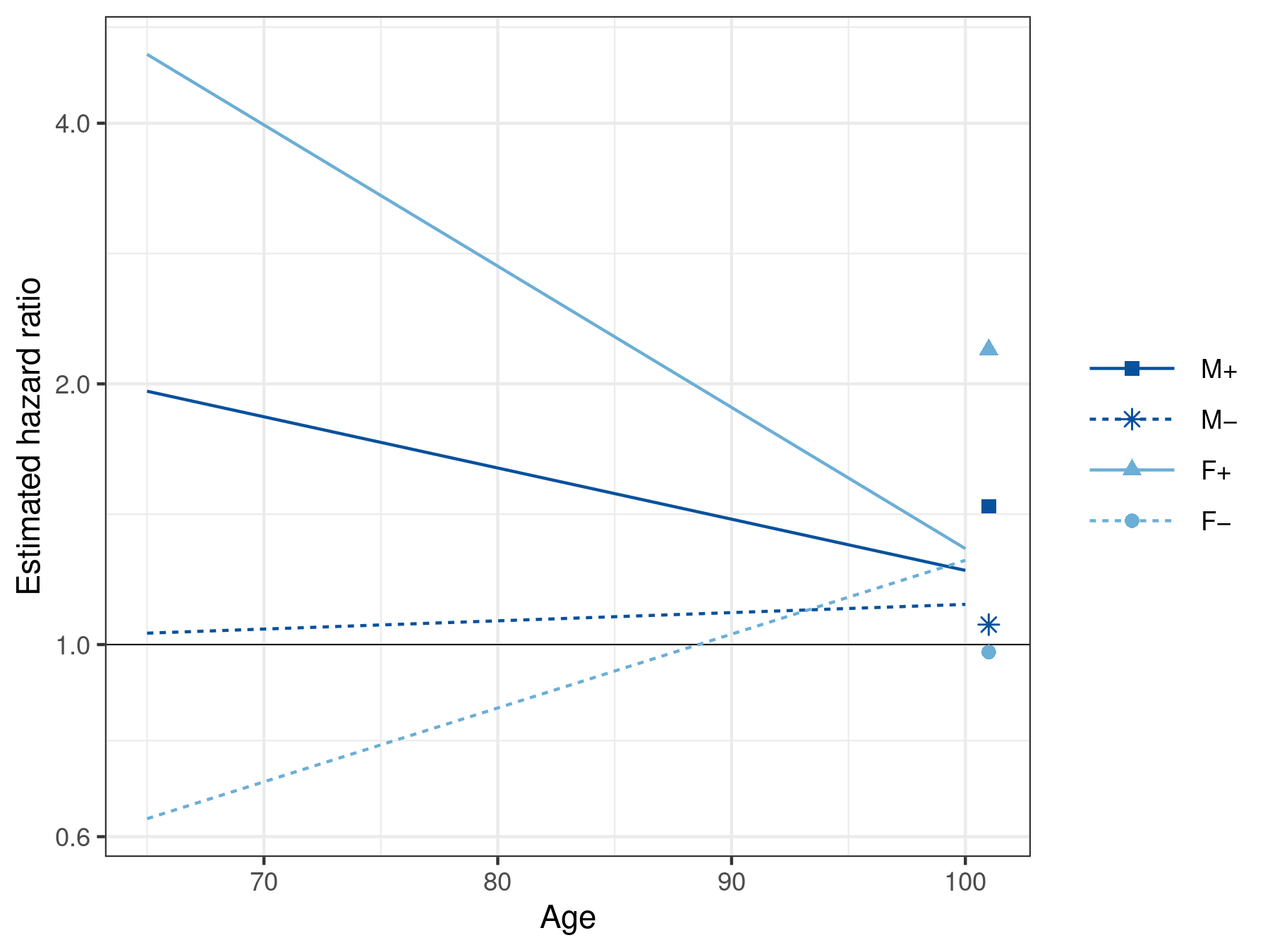


**Supplementary Figure 5. Hazard ratios for the without dementia to dementia transition by age for the APOE and sex predictor variable subgroups.** The lines represent the estimate hazard ratio at each age from the model with an age-dependent APOE genotype covariate (i.e. age × APOE interaction). Because the model also included an APOE × sex interaction, estimates are shown for the combination of these four predictor variable subgroups. Hazard ratios were referenced to the overall study population average and are shown for the moderate amyloid PET group. As a reference, points at the right side of the figure indicate the hazard ratios from the main model without an age-dependent APOE covariate which are shown in Supplementary Table 2a. + and – symbols represent APOE ε4 status: + refers to carrier, - refers to non-carrier.


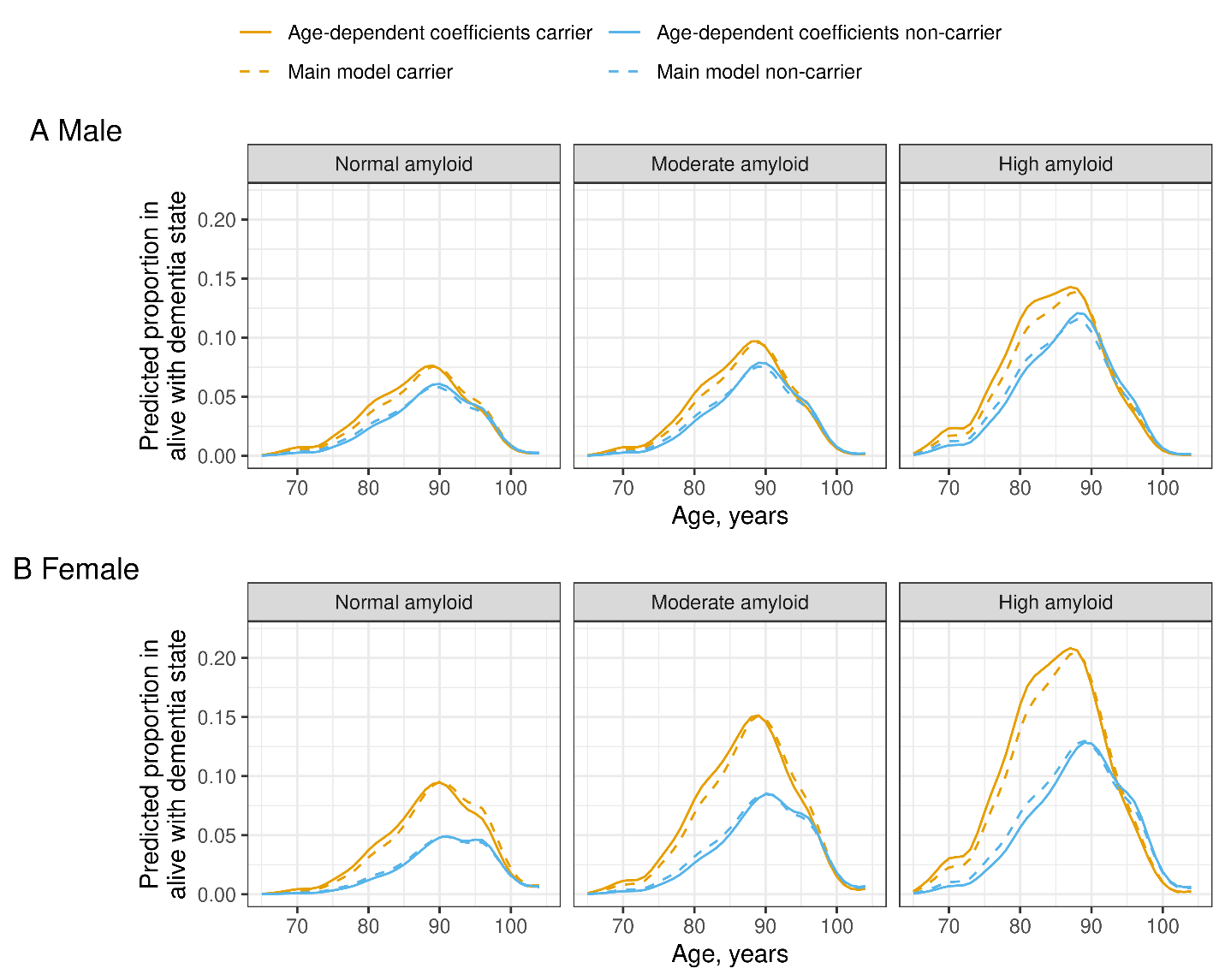


**Supplementary Figure 6. Comparison of the predicted proportion in the alive with dementia state by age between the age constant model (main model, dashed lines) vs the model with age varying hazards for CMC and APOE (age-dependent coefficients, solid lines).** All estimates are shown assuming a starting age of 65. The rows and columns illustrate the different sex and amyloid PET risk predictor variable subgroups. APOE ε4 subgroups are represented by different colors. Other predictor variable effects were weighted to the frequencies observed in the overall study population. The impact of age-dependent effects on the predicted proportion in the alive with dementia state by age is negligible.


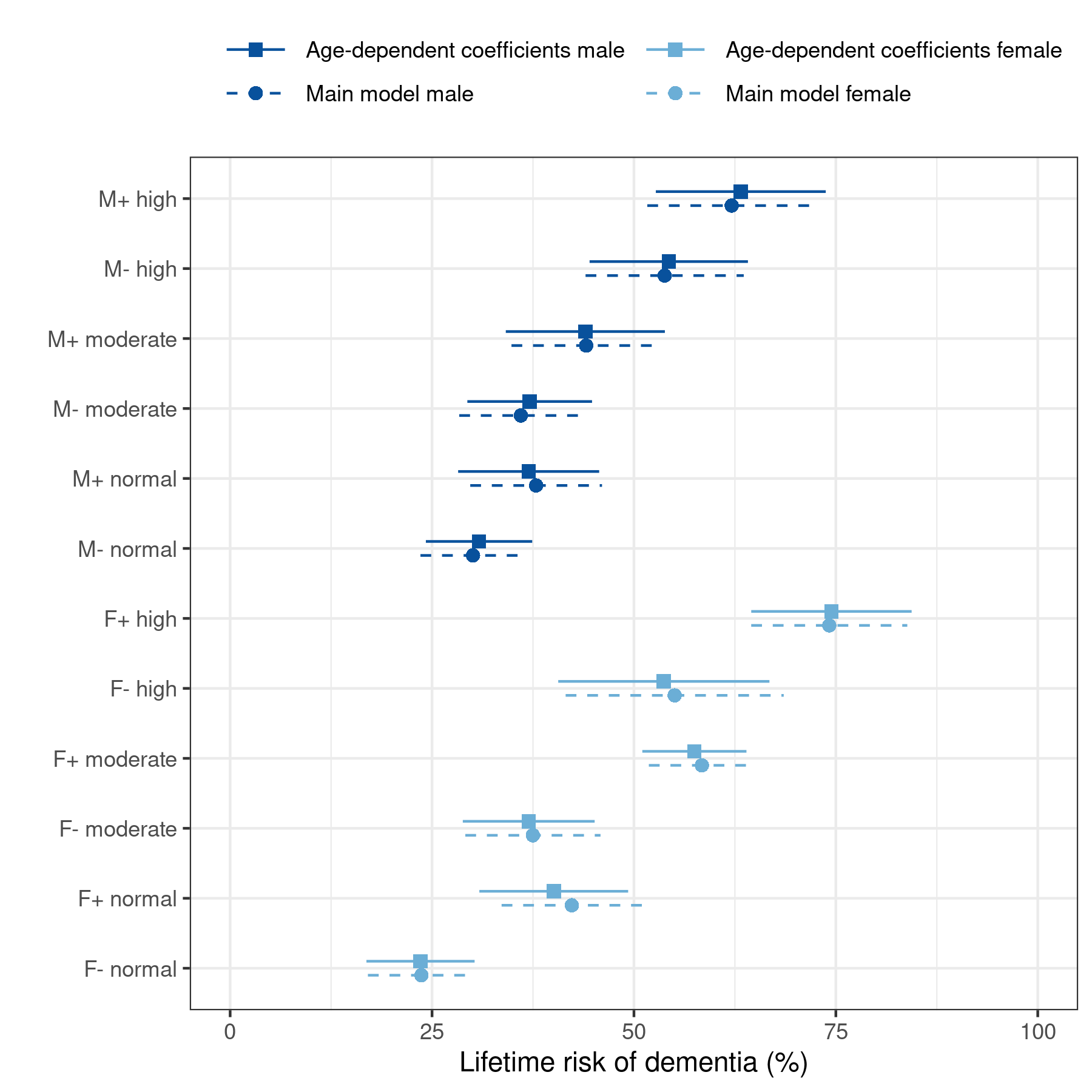


**Supplementary Figure 7. Comparison of the remaining lifetime risk of ever experiencing dementia between the age constant model (main model, dashed lines) vs the model with age varying hazards for CMC and APOE (age-dependent coefficients, solid lines).** The remaining lifetime risk of dementia estimates are shown by sex, APOE genotype, and amyloid group for a 65 year old person without dementia and are averaged over the combinations of education and CMC. The 95% confidence interval was computed using a 20-fold grouped jack-knife standard error. The impact of age-dependent effects on the estimated lifetime risk of dementia is negligible. + and – symbols in the top panel represent APOE ε4 status: + refers to carrier, - refers to non-carrier.

REFERNCES

1. Roberts, R.O., et al., *The Mayo Clinic Study of Aging: design and sampling, participation, baseline measures and sample characteristics.* Neuroepidemiology, 2008. **30**(1): p. 58-69.

2. St Sauver, J.L., et al., *Generalizability of epidemiological findings and public health decisions: an illustration from the Rochester Epidemiology Project.* Mayo Clinic Proceedings, 2012. **87**(2): p. 151-160.

3. Schemper, M. and T.L. Smith, *A note on quantifying follow-up in studies of failure time.* Control Clin Trials, 1996. **17**(4): p. 343-6.

4. Knopman, D.S., et al., *Passive case-finding for Alzheimer's disease and dementia in two U.S. communities.* Alzheimer's & dementia : the journal of the Alzheimer's Association, 2011. **7**(1): p. 53-60.

5. Rocca, W.A., et al., *Prevalence of multimorbidity in a geographically defined American population: patterns by age, sex, and race/ethnicity.* Mayo Clin Proc, 2014. **89**(10): p. 1336-49.

6. Vemuri, P., et al., *Age, vascular health, and Alzheimer disease biomarkers in an elderly sample.* Ann Neurol, 2017. **82**(5): p. 706-718.

7. Jack, C.R., et al., *Defining imaging biomarker cut points for brain aging and Alzheimer's disease.* Alzheimer's & dementia : the journal of the Alzheimer's Association, 2017. **13**(3): p. 205-216.

8. Klunk, W.E., et al., *The Centiloid Project: Standardizing quantitative amyloid plaque estimation by PET.* Alzheimer's & dementia, 2015. **11**(1): p. 1-15.

9. Schwarz, C.G., et al., *Considerations for Performing Level-2 Centiloid Transformations for Amyloid PET SUVR values.* Sci Rep, 2018. **8**(1): p. 7421.

10. Putter, H., M. Fiocco, and R.B. Geskus, *Tutorial in biostatistics: competing risks and multi-state models.* Statistics in medicine, 2007. **26**(11): p. 2389-2430.

11. Therneau, T.M. and P.M. Grambsch, *Modeling survival data : extending the Cox model*. Statistics for biology and health. 2000, New York: Springer. xiii, 350 p.

12. Naimi, A.I., S.R. Cole, and E.H. Kennedy, *An introduction to g methods.* Int J Epidemiol, 2017. **46**(2): p. 756-762.

13. Grambsch, P.M. and T.M. Therneau, *Proportional Hazards Tests and Diagnostics Based on Weighted Residuals.* Biometrika, 1994. **81**(3): p. 515-526.
